# Supplementary material for: Reduced IQGAP2 Promotes Bladder Cancer through Regulation of MAPK/ERK Pathway and Cytokines
Source: Int J Mol Sci. 2022 Nov 4;23(21):13508. doi: 10.3390/ijms232113508 (PMC9655856; doi:10.3390/ijms232113508)
Supplement: Supplementary file 1 [file ijms-23-13508-s001.zip › ijms-1975198-supplementary.pdf]

Supplementary Data

Table S1

Table S2

Table S3

Figure S1

Figure S2

Figure S3

Table S1. Sequences of primers for Expression analyses of target genes by RT-qPCR

Target gene	Primer	Sequence (5' – 3')
RPS13	forward	CGAAAGCATCTTGAGAGGAACA
	reverse	TCGAGCCAAACGGTGAATC
RPS23	forward	TGGAGGTGCTTCTCATGCAA
	reverse	AATGGCAGAATTTGGCTGTTTG
IQGAP2	forward	TCAAGATTGGACTGC
	reverse	AGGTTTGGTCTGGAGGAG
CDH1	forward	CGTAGCAGTGACGAATGTGGT
	reverse	TGGGGGCTTCATTCACATCC
CDH2	forward	TGACTATGAAGGCAGTGGCTC
	reverse	CCTGCTCACCACCACTACTT
MMP9	forward	GAGACGGGTATCCCTTCGAC
	reverse	AGTTGGAACCACGACGCC
IQGAP2 TV1	forward	AGGCGGCAGAACATTGCTTA
	reverse	CCAATTCAGTGGTTGGTGGC
IQGAP2 TV2	forward	GTGTAGGAAGGAGTTGTGGCA
	reverse	TCGCTCTTAACCACTGGACG
IQGAP2 TV3+4	forward	GAGTTCCTGGGTCACACCTG
	reverse	TCCTTTCCAAAAAGCCTCGAT
IL6	forward	TGCGTTGCACTTGTTTACGC
	reverse	GGAGGCTACCACTTCCACCT
IL8	forward	ACCGGAAGGAACCATCTCAC
	reverse	GGCAAAACTGCACCTTCACAC
CCL2	forward	GCTCAGCCAGATGCAATCAAT
	reverse	CACTTGCTGCTGGTGATTCTT
BCL-2	forward	CCTTTGTGGAACTGTACGGC
	reverse	CCGGCCAACAACATGGAAAG
CASPASE3	forward	TCTGGTTTTCGGTGGGTGTG
	reverse	GTCGGCCTCCACTGGTATTT

Table S2. The Significant Changes of IQGAP2 Expression in Transcription Level between bladder cancer and normal tissues (ONCOMINE Database)

Types of BC VS. Normal tissues	Fold Change	P value	t-test	Reference or Source
Bladder Urothelial Carcinoma	-1.124	0.013	-2.526	TCGA [42]
Infiltrating Bladder Urothelial Carcinoma, Micropapillary Variant	-1.135	2.51E-5	-4.586	TCGA [42]
Infiltrating Bladder Urothelial Carcinoma	-1.121	6.98E-9	-6.190	TCGA [42]
Superficial Bladder Cancer	-1.303	1.95E-10	-6.881	Lee [43]
Infiltrating Bladder Urothelial Carcinoma	-1.169	9.82E-4	-3.162	Lee [43]
Superficial Bladder Cancer	-3.363	1.91E-9	-7.671	Sanchez-Carbayo [44]
Infiltrating Bladder Urothelial Carcinoma	-1.504	7.91E-6	-4.496	Sanchez-Carbayo [44]
Superficial Bladder Cancer	-1.324	0.034	-1.885	Dyrskjot [45]

Table S3. The correlated frequently altered neighbor genes of IQGAP2 in patients with Bladder Cancer (TCGA Database) (Spearman's Correlation > 0.5)

Correlated Gene	Cytoband	Spearman's Correlation	p-Value	q-Value
TNFSF8	9q32-q33.1	0,808337256	1,87E-95	3,74E-91
SLCO2B1	11q13.4	0,801996451	6,84E-93	6,85E-89
CD84	1q23.3	0,793993569	8,72E-90	5,82E-86
EVI2A	17q11.2	0,784810509	2,18E-86	1,09E-82
MPEG1	11q12.1	0,781185079	4,30E-85	1,72E-81
RCSD1	1q24.2	0,780779407	5,98E-85	2,00E-81
DOCK2	5q35.1	0,77991753	1,20E-84	3,44E-81
NCKAP1L	12q13.13-q13.2	0,774234585	1,12E-82	2,80E-79
IL10RA	11q23.3	0,770261613	2,47E-81	5,48E-78
PIK3R5	17p13.1	0,768106391	1,29E-80	2,57E-77
ZEB2	2q22.3	0,766308959	5,02E-80	8,95E-77
BTK	Xq22.1	0,766221322	5,37E-80	8,95E-77
C3AR1	12p13.31	0,764845288	1,51E-79	2,33E-76
MS4A4A	11q12.2	0,764159568	2,52E-79	3,61E-76
WIPF1	2q31.1	0,763299104	4,79E-79	6,40E-76
EVI2B	17q11.2	0,762269376	1,03E-78	1,29E-75
MS4A6A	11q12.2	0,761587719	1,70E-78	2,00E-75
PTPRC	1q31.3-q32.1	0,761283288	2,13E-78	2,37E-75
TFEC	7q31.2	0,761196182	2,27E-78	2,39E-75
PLEK	2p14	0,76079475	3,05E-78	3,05E-75
CYBB	Xp21.1-p11.4	0,760529897	3,71E-78	3,53E-75
TLR8	Xp22.2	0,760077578	5,17E-78	4,70E-75
WDFY4	10q11.23	0,759672614	6,95E-78	6,04E-75
TASL	Xp21.2	0,758820353	1,29E-77	1,08E-74
GGTA1	9q33.2	0,758375857	1,79E-77	1,43E-74
CD28	2q33.2	0,75755304	3,24E-77	2,50E-74
DOCK10	2q36.2	0,756843013	5,41E-77	4,01E-74
FGL2	7q11.23	0,754484038	2,93E-76	2,10E-73
AOAH	7p14.2	0,753287342	6,87E-76	4,74E-73
IRF8	16q24.1	0,75165034	2,18E-75	1,45E-72
ITGB2	21q22.3	0,75107982	3,25E-75	2,10E-72
NFAM1	22q13.2	0,749345818	1,09E-74	6,82E-72
CD163	12p13.31	0,748374219	2,14E-74	1,30E-71
CD53	1p13.3	0,747363396	4,30E-74	2,53E-71
MSR1	8p22	0,746235606	9,33E-74	5,34E-71
ITGA4	2q31.3	0,745824633	1,24E-73	6,87E-71
GPR34	Xp11.4	0,742153618	1,49E-72	8,05E-70
SLA	8q24.22	0,741768264	1,93E-72	1,02E-69
KLHL6	3q27.1	0,741360825	2,53E-72	1,30E-69
LCP2	5q35.1	0,740510201	4,47E-72	2,24E-69
GIMAP4	7q36.1	0,740091156	5,92E-72	2,89E-69
CD37	19q13.33	0,739893091	6,75E-72	3,22E-69
LAIR1	19q13.42	0,739192882	1,07E-71	5,00E-69
BIN2	12q13.13	0,736639618	5,78E-71	2,63E-68
SELPLG	12q24.11	0,734292659	2,67E-70	1,19E-67
GNG2	14q22.1	0,73421792	2,80E-70	1,22E-67
RNASE6	14q11.2	0,732757727	7,20E-70	3,06E-67
PIK3AP1	10q24.1	0,731525395	1,59E-69	6,62E-67
RASGRP4	19q13.2	0,73125133	1,89E-69	7,73E-67

MNDA	1q23.1	0,730837201	2,46E-69	9,86E-67
DOCK11	Xq24	0,730365271	3,33E-69	1,31E-66
SIGLEC7	19q13.41	0,729332638	6,42E-69	2,43E-66
CMKLR1	12q23.3	0,729326001	6,44E-69	2,43E-66
PIK3R6	17p13.1	0,728772619	9,15E-69	3,39E-66
PARVG	22q13.31	0,728456206	1,12E-68	4,07E-66
CYTIP	2q24.1	0,728072057	1,42E-68	5,09E-66
IKZF1	7p12.2	0,727392344	2,18E-68	7,67E-66
PPP1R16B	20q11.23	0,727024659	2,75E-68	9,49E-66
CR1	1q32.2	0,72683887	3,09E-68	1,05E-65
ITM2A	Xq21.1	0,726746378	3,28E-68	1,09E-65
SLC7A7	14q11.2	0,725746156	6,12E-68	2,01E-65
LY86	6p25.1	0,72520111	8,60E-68	2,78E-65
CD33	19q13.41	0,724885636	1,05E-67	3,32E-65
SCIMP	17p13.2	0,724665417	1,20E-67	3,75E-65
SAMSN1	21q11.2	0,723837588	2,00E-67	6,17E-65
CSF2RB	22q12.3	0,723355411	2,70E-67	8,19E-65
LILRB4	19q13.42	0,722019958	6,14E-67	1,83E-64
MRC1	10p12.33	0,721840678	6,86E-67	2,02E-64
GPR183	13q32.3	0,721495432	8,47E-67	2,46E-64
STARD8	Xq13.1	0,721469813	8,61E-67	2,46E-64
GAB3	Xq28	0,719773976	2,42E-66	6,78E-64
PDE4B	1p31.3	0,719749946	2,46E-66	6,78E-64
GPR65	14q31.3	0,719739168	2,47E-66	6,78E-64
CSF1R	5q32	0,719021114	3,82E-66	1,03E-63
VCAM1	1p21.2	0,718837714	4,27E-66	1,14E-63
IGSF6	16p12.2	0,718341401	5,77E-66	1,52E-63
PILRA	7q22.1	0,718111532	6,63E-66	1,72E-63
WAS	Xp11.23	0,718095277	6,69E-66	1,72E-63
C1QC	1p36.12	0,717676353	8,62E-66	2,18E-63
IFFO1	12p13.31	0,716316076	1,95E-65	4,88E-63
IL2RA	10p15.1	0,715751228	2,73E-65	6,75E-63
TYROBP	19q13.12	0,715686333	2,84E-65	6,93E-63
HAVCR2	5q33.3	0,714355531	6,27E-65	1,51E-62
CD48	1q23.3	0,714050393	7,52E-65	1,79E-62
FPR3	19q13.41	0,713869643	8,37E-65	1,97E-62
SPI1	11p11.2	0,71381063	8,67E-65	2,02E-62
FCGR3A	1q23.3	0,713594012	9,85E-65	2,27E-62
ATP8B4	15q21.2	0,713072963	1,34E-64	3,05E-62
SASH3	Xq26.1	0,711798347	2,84E-64	6,39E-62
LILRB1	19q13.42	0,710574471	5,82E-64	1,29E-61
CYTH4	22q13.1	0,710227957	7,12E-64	1,57E-61
SLAMF8	1q23.2	0,709724224	9,55E-64	2,08E-61
PLXNC1	12q22	0,709352957	1,18E-63	2,55E-61
AP1S2	Xp22.2	0,709278974	1,24E-63	2,63E-61
C5AR1	19q13.32	0,709131971	1,35E-63	2,84E-61
LILRB2	19q13.4	0,70853	1,91E-63	3,98E-61
FLI1	11q24.3	0,707558167	3,35E-63	6,91E-61
RUBCNL	13q14.13	0,707406607	3,65E-63	7,46E-61
LRRC25	19p13.11	0,707319168	3,84E-63	7,77E-61
VSIG4	Xq12	0,70715856	4,22E-63	8,44E-61
SYT11	1q22	0,706461885	6,29E-63	1,25E-60
P2RY8	Xp22.33 and Yp11.3	0,706380432	6,59E-63	1,29E-60
ITK	5q33.3	0,706347901	6,72E-63	1,30E-60
SIGLEC10	19q13.41	0,706040134	8,01E-63	1,54E-60

HCK	20q11.21	0,70484432	1,59E-62	3,02E-60
RGS18	1q31.2	0,704312533	2,15E-62	4,06E-60
C1QB	1p36.12	0,704161956	2,34E-62	4,38E-60
FCER1G	1q23.3	0,703435629	3,53E-62	6,55E-60
PTPN22	1p13.2	0,703057934	4,38E-62	8,02E-60
SIGLEC9	19q13.3-q13.4	0,703045962	4,41E-62	8,02E-60
GPR141	7p14.1	0,702806575	5,05E-62	9,10E-60
AIF1	6p21.33	0,702420534	6,28E-62	1,12E-59
SLAMF6	1q23.2-q23.3	0,702365912	6,47E-62	1,15E-59
KCNA3	1p13.3	0,702186248	7,16E-62	1,26E-59
TMEM176B	7q36.1	0,701249986	1,21E-61	2,11E-59
LILRB5	19q13.4	0,701235882	1,22E-61	2,11E-59
GYPC	2q14.3	0,701094003	1,32E-61	2,26E-59
SPN	16p11.2	0,700624693	1,72E-61	2,92E-59
CD180	5q12.3	0,700529635	1,82E-61	3,06E-59
CPVL	7p14.3	0,699913883	2,56E-61	4,27E-59
ABCD2	12q12	0,699729711	2,84E-61	4,70E-59
CCR2	3p21.31	0,699493214	3,24E-61	5,32E-59
LYZ	12q15	0,698814363	4,73E-61	7,70E-59
ITGAX	16p11.2	0,698096839	7,05E-61	1,14E-58
CD226	18q22.2	0,697403934	1,03E-60	1,66E-58
SIGLEC1	20p13	0,69635206	1,85E-60	2,93E-58
PLEKHO2	15q22.31	0,695968297	2,28E-60	3,59E-58
MYO1F	19p13.2	0,695804508	2,50E-60	3,90E-58
LAX1	1q32.1	0,69563618	2,74E-60	4,25E-58
ITGAM	16p11.2	0,695463857	3,01E-60	4,63E-58
ITGAL	16p11.2	0,694857999	4,19E-60	6,40E-58
C1QA	1p36.12	0,694303557	5,67E-60	8,60E-58
FERMT3	11q13.1	0,694060259	6,48E-60	9,75E-58
CD209	19p13.2	0,693990645	6,73E-60	1,00E-57
MPP1	Xq28	0,693816432	7,40E-60	1,10E-57
LILRA6	19q13.42	0,692911526	1,21E-59	1,78E-57
SNX20	16q12.1	0,692072478	1,91E-59	2,78E-57
GIMAP1	7q36.1	0,692062112	1,92E-59	2,78E-57
FCGR2C	1q23.3	0,690625827	4,16E-59	5,99E-57
CELF2	10p14	0,690342775	4,84E-59	6,92E-57
DOK2	8p21.3	0,690268214	5,04E-59	7,13E-57
TBXAS1	7q34	0,690259556	5,06E-59	7,13E-57
LAT2	7q11.23	0,688968684	1,01E-58	1,41E-56
SH2B3	12q24.12	0,68884412	1,08E-58	1,50E-56
TMEM150B	19q13.42	0,688796936	1,11E-58	1,53E-56
FOLR2	11q13.4	0,687220725	2,56E-58	3,51E-56
P2RX7	12q24.31	0,687185912	2,61E-58	3,55E-56
VIM	10p13	0,685251907	7,24E-58	9,79E-56
LY96	8q21.11	0,685131584	7,71E-58	1,04E-55
ARHGAP31	3q13.32-q13.33	0,685019506	8,18E-58	1,09E-55
CCR4	3p22.3	0,684461304	1,10E-57	1,45E-55
FCGR1A	1q21.2	0,683960006	1,43E-57	1,88E-55
GVINP1	11p15.4	0,683592114	1,73E-57	2,26E-55
P2RY13	3q25.1	0,68321324	2,11E-57	2,74E-55
FCGR2A	1q23.3	0,682786601	2,63E-57	3,40E-55
IL21R	16p12.1	0,682670283	2,80E-57	3,59E-55
C1ORF162	1p13.2	0,68216973	3,63E-57	4,62E-55
ANXA6	5q33.1	0,682015011	3,93E-57	4,98E-55
FPR1	19q13.41	0,681860587	4,26E-57	5,36E-55

ARHGAP15	2q22.2-q22.3	0,681151367	6,15E-57	7,69E-55
FAM78A	9q34.13	0,680664242	7,91E-57	9,83E-55
CD4	12p13.31	0,680301152	9,54E-57	1,18E-54
CD300C	17q25.1	0,680271924	9,68E-57	1,19E-54
CD86	3q13.33	0,679909438	1,17E-56	1,42E-54
ZC3H12D	6q25.1	0,679646122	1,34E-56	1,62E-54
P2RY10	Xq21.1	0,67934031	1,56E-56	1,89E-54
GIMAP5	7q36.1	0,678604431	2,28E-56	2,73E-54
SLAMF1	1q23.3	0,678311279	2,65E-56	3,16E-54
NRROS	3q29	0,677830544	3,39E-56	4,01E-54
FCGR1BP	1p11.2	0,677771296	3,49E-56	4,11E-54
RHOH	4p14	0,677418806	4,18E-56	4,89E-54
HLA-DPB1	6p21.32	0,67669816	6,03E-56	7,02E-54
CCR5	3p21.31	0,676479953	6,74E-56	7,80E-54
NLRP3	1q44	0,675889054	9,10E-56	1,05E-53
TAGAP	6q25.3	0,675543161	1,08E-55	1,24E-53
RASSF4	10q11.21	0,674747718	1,62E-55	1,84E-53
CD300LF	17q25.1	0,673676821	2,78E-55	3,14E-53
TMEM273	10q11.23	0,673569602	2,93E-55	3,29E-53
ZNF831	20q13.32	0,672922032	4,05E-55	4,53E-53
FCGR2B	1q23.3	0,672584192	4,80E-55	5,34E-53
DDR2	1q23.3	0,672158584	5,94E-55	6,57E-53
DAB2	5p13.1	0,672060493	6,24E-55	6,86E-53
HLA-DMB	6p21.32	0,672000596	6,43E-55	7,03E-53
GIMAP7	7q36.1	0,671949711	6,59E-55	7,17E-53
CXCR4	2q22.1	0,671938756	6,63E-55	7,17E-53
IL7R	5p13.2	0,671890294	6,79E-55	7,31E-53
TNFSF4	1q25.1	0,671667896	7,59E-55	8,12E-53
SH2D1A	Xq25	0,671479434	8,33E-55	8,87E-53
IL10	1q32.1	0,671340325	8,93E-55	9,46E-53
ARHGAP9	12q13.3	0,67111363	1,00E-54	1,05E-52
PIP4K2A	10p12.2	0,670525971	1,34E-54	1,40E-52
FCGR1CP	1q21.1	0,670119905	1,64E-54	1,71E-52
ZEB1	10p11.22	0,67010104	1,65E-54	1,71E-52
CD5	11q12.2	0,670091499	1,66E-54	1,71E-52
MYO1G	7p13	0,669911809	1,81E-54	1,86E-52
TNFAIP8L2	1q21.3	0,668896215	3,00E-54	3,06E-52
CST7	20p11.21	0,668705393	3,29E-54	3,34E-52
THEMIS2	1p35.3	0,668504501	3,63E-54	3,67E-52
LILRB3	19q13.42	0,667952885	4,77E-54	4,79E-52
ENTPD1	10q24.1	0,667875438	4,95E-54	4,95E-52
STAB1	3p21.1	0,667638737	5,56E-54	5,54E-52
HLA-DQA1	6p21.32	0,6672237	6,82E-54	6,75E-52
NCF1B	7q11.23	0,666784311	8,45E-54	8,33E-52
NCF1	7q11.23	0,666485475	9,78E-54	9,59E-52
FAM20A	17q24.2	0,665742361	1,41E-53	1,37E-51
ABI3	17q21.32	0,665194809	1,83E-53	1,78E-51
GZMK	5q11.2	0,665132885	1,89E-53	1,83E-51
P2RY14	3q25.1	0,664642427	2,40E-53	2,31E-51
BTLA	3q13.2	0,663888522	3,45E-53	3,31E-51
MFNG	22q13.1	0,663206026	4,80E-53	4,58E-51
NCF1C	7q11.23	0,663100705	5,05E-53	4,79E-51
RTN1	14q23.1	0,661555361	1,06E-52	1,00E-50
TMEM176A	7q36.1	0,661510719	1,08E-52	1,02E-50
CASS4	20q13.31	0,661475556	1,10E-52	1,03E-50

ADORA3	1p13.2	0,661133964	1,30E-52	1,21E-50
OLFML3	1p13.2	0,660934898	1,43E-52	1,32E-50
HLA-DPA1	6p21.32	0,660491591	1,77E-52	1,63E-50
CCR8	3p22.1	0,66020215	2,03E-52	1,86E-50
SAMD3	6q23.1	0,660169891	2,06E-52	1,88E-50
LY9	1q23.3	0,659986935	2,25E-52	2,04E-50
CD300LB	17q25.1	0,65982574	2,42E-52	2,20E-50
HCST	19q13.12	0,659329524	3,07E-52	2,77E-50
GRAP2	22q13.1	0,6589525	3,67E-52	3,29E-50
CXCR3	Xq13.1	0,658684499	4,17E-52	3,72E-50
CEACAM21	19q13.2	0,658511003	4,53E-52	4,03E-50
LST1	6p21.33	0,658479132	4,60E-52	4,07E-50
FMNL3	12q13.12	0,658416232	4,73E-52	4,17E-50
PLA2G7	6p12.3	0,658348737	4,89E-52	4,29E-50
COL15A1	9q22.33	0,657657363	6,78E-52	5,92E-50
RECK	9p13.3	0,65757167	7,06E-52	6,14E-50
CLEC4E	12p13.31	0,657172097	8,52E-52	7,38E-50
TNFRSF1B	1p36.22	0,65706246	8,97E-52	7,74E-50
SLC24A4	14q32.12	0,656876196	9,79E-52	8,41E-50
LRRK2	12q12	0,656551659	1,14E-51	9,76E-50
F2R	5q13.3	0,656516852	1,16E-51	9,87E-50
ATP10A	15q12	0,6561474	1,38E-51	1,17E-49
ECM2	9q22.31	0,656087444	1,42E-51	1,20E-49
GAS7	17p13.1	0,656021953	1,46E-51	1,23E-49
F13A1	6p25.1	0,655626705	1,76E-51	1,47E-49
PLCL1	2q33.1	0,655589718	1,79E-51	1,49E-49
TM6SF1	15q25.2	0,655109043	2,24E-51	1,86E-49
CD38	4p15.32	0,654412804	3,10E-51	2,57E-49
CD14	5q31.3	0,653836341	4,06E-51	3,34E-49
CLEC4A	12p13.31	0,653807658	4,12E-51	3,38E-49
HLA-DRA	6p21.32	0,651534999	1,18E-50	9,63E-49
RGS1	1q31.2	0,651184807	1,39E-50	1,13E-48
RASAL3	19p13.12	0,651144876	1,41E-50	1,14E-48
PTPN7	1q32.1	0,651136218	1,42E-50	1,14E-48
TLR7	Xp22.2	0,651104531	1,44E-50	1,16E-48
NID2	14q22.1	0,651078088	1,46E-50	1,17E-48
CD52	1p36.11	0,650386714	2,00E-50	1,59E-48
GNGT2	17q21.32	0,649775407	2,65E-50	2,10E-48
GLIPR2	9p13.3	0,649567949	2,91E-50	2,30E-48
TREM2	6p21.1	0,647374032	7,91E-50	6,24E-48
ADPRH	3q13.33	0,646888321	9,86E-50	7,74E-48
CLEC10A	17p13.1	0,646706001	1,07E-49	8,38E-48
CNRIP1	2p14	0,646659512	1,09E-49	8,52E-48
MS4A7	11q12.2	0,646645377	1,10E-49	8,54E-48
FOXP3	Xp11.23	0,646589368	1,13E-49	8,73E-48
FMNL1	17q21.31	0,645677312	1,71E-49	1,31E-47
HSD11B1	1q32.2	0,645606603	1,76E-49	1,35E-47
KCNMA1	10q22.3	0,645529955	1,82E-49	1,39E-47
CD80	3q13.33	0,645384836	1,95E-49	1,48E-47
CCR1	3p21.31	0,645221284	2,09E-49	1,59E-47
TNFSF13B	13q33.3	0,644919914	2,40E-49	1,81E-47
MEF2C	5q14.3	0,644863964	2,46E-49	1,85E-47
PYHIN1	1q23.1	0,644739859	2,60E-49	1,95E-47
ALOX5AP	13q12.3	0,644226892	3,28E-49	2,45E-47
LILRA1	19q13.42	0,64391508	3,77E-49	2,80E-47

NLRC4	2p22.3	0,643604249	4,33E-49	3,20E-47
ELMO1	7p14.2-p14.1	0,643600362	4,34E-49	3,20E-47
HK3	5q35.2	0,643348111	4,86E-49	3,57E-47
RSPO3	6q22.33	0,643141254	5,33E-49	3,90E-47
CD27	12p13.31	0,643055461	5,53E-49	4,04E-47
IGF1	12q23.2	0,642630382	6,69E-49	4,87E-47
FBN1	15q21.1	0,642034036	8,73E-49	6,33E-47
LILRA2	19q13.4	0,64124223	1,24E-48	8,96E-47
C5AR2	19q13.32	0,641073186	1,34E-48	9,62E-47
TIGIT	3q13.31	0,64036367	1,83E-48	1,31E-46
ADAMTSL1	9p22.2-p22.1	0,640210836	1,96E-48	1,40E-46
ADAMDEC1	8p21.2	0,639147367	3,13E-48	2,23E-46
ADAP2	17q11.2	0,638884246	3,52E-48	2,49E-46
CD93	20p11.21	0,638234923	4,68E-48	3,31E-46
PRAM1	19p13.2	0,637581006	6,23E-48	4,39E-46
IRF4	6p25.3	0,637460153	6,57E-48	4,61E-46
GPRIN3	4q22.1	0,637382555	6,79E-48	4,75E-46
CD300A	17q25.1	0,636408515	1,04E-47	7,25E-46
GPC6	13q31.3-q32.1	0,635350869	1,65E-47	1,14E-45
TXLNB	6q24.1	0,635061142	1,87E-47	1,29E-45
COLEC12	18p11.32	0,634803788	2,09E-47	1,44E-45
SLIT2	4p15.31	0,634792258	2,10E-47	1,44E-45
PDGFRB	5q32	0,634731937	2,15E-47	1,48E-45
ARHGAP25	2p13.3	0,634692503	2,19E-47	1,50E-45
GIMAP8	7q36.1	0,634420262	2,46E-47	1,68E-45
OLFML1	11p15.4	0,633908577	3,07E-47	2,09E-45
CXCL12	10q11.21	0,63333682	3,93E-47	2,66E-45
NCF2	1q25.3	0,632631487	5,33E-47	3,59E-45
CCDC170	6q25.1	0,632466518	5,72E-47	3,84E-45
CD3E	11q23.3	0,632451267	5,76E-47	3,85E-45
PIK3CG	7q22.3	0,632152518	6,55E-47	4,37E-45
HGF	7q21.11	0,631939833	7,17E-47	4,77E-45
PCED1B-AS1	12q13.11	0,631793408	7,64E-47	5,06E-45
CERKL	2q31.3	0,631778975	7,68E-47	5,07E-45
C1S	12p13.31	0,631088484	1,03E-46	6,80E-45
DOCK4	7q31.1	0,630940951	1,10E-46	7,21E-45
CCDC80	3q13.2	0,63078211	1,18E-46	7,70E-45
CSF2RA	Xp22.32 and Yp11.3	0,630238622	1,48E-46	9,67E-45
ZNF521	18q11.2	0,630192507	1,51E-46	9,83E-45
COL6A3	2q37.3	0,629268083	2,24E-46	1,45E-44
NEXN	1p31.1	0,629130119	2,38E-46	1,53E-44
GPR171	3q25.1	0,62764209	4,46E-46	2,87E-44
KCNN3	1q21.3	0,627446988	4,84E-46	3,11E-44
CALHM5	6q22.1	0,627333366	5,08E-46	3,25E-44
CLEC4D	12p13.31	0,627289554	5,18E-46	3,30E-44
TRPV2	17p11.2	0,627126643	5,55E-46	3,52E-44
BNC2	9p22.3-p22.2	0,626851189	6,23E-46	3,94E-44
TRAT1	3q13.13	0,62662808	6,84E-46	4,32E-44
ATP8B2	1q21.3	0,625463493	1,11E-45	7,02E-44
IL12RB1	19p13.11	0,625370318	1,16E-45	7,27E-44
CD2	1p13.1	0,624986467	1,36E-45	8,51E-44
NLRC3	16p13.3	0,624864349	1,43E-45	8,93E-44
PMP22	17p12	0,624650558	1,57E-45	9,73E-44
PDCD1LG2	9p24.1	0,624535646	1,64E-45	1,02E-43
ITGA9	3p22.2	0,624341004	1,78E-45	1,10E-43

S1PR1	1p21.2	0,624078801	1,99E-45	1,22E-43
JAKMIP1	4p16.1	0,623748396	2,28E-45	1,40E-43
ADAMTS2	5q35.3	0,623629487	2,40E-45	1,47E-43
MAP4K1	19q13.2	0,623466052	2,57E-45	1,57E-43
SIT1	9p13.3	0,623439436	2,59E-45	1,58E-43
HLA-DOA	6p21.32	0,623235415	2,82E-45	1,71E-43
VNN1	6q23.2	0,62303824	3,06E-45	1,85E-43
BICC1	10q21.1	0,62277514	3,42E-45	2,06E-43
PREX1	20q13.13	0,622559827	3,74E-45	2,25E-43
EGR2	10q21.3	0,622230837	4,28E-45	2,57E-43
FCRL3	1q23.1	0,622169312	4,39E-45	2,62E-43
NRP2	2q33.3	0,622021286	4,67E-45	2,78E-43
C1R	12p13.31	0,621615438	5,52E-45	3,28E-43
FCRL6	1q23.2	0,621567175	5,63E-45	3,34E-43
IL16	15q25.1	0,621468081	5,87E-45	3,46E-43
CD200R1	3q13.2	0,621312613	6,26E-45	3,68E-43
GEM	8q22.1	0,620781301	7,79E-45	4,57E-43
CCDC141	2q31.2	0,620568915	8,50E-45	4,97E-43
BEST1	11q12.3	0,620542271	8,59E-45	5,01E-43
CYSLTR1	Xq21.1	0,620058168	1,05E-44	6,10E-43
PALD1	10q22.1	0,619897691	1,12E-44	6,50E-43
THEMIS	6q22.33	0,61984894	1,14E-44	6,61E-43
FCN1	9q34.3	0,619748082	1,19E-44	6,87E-43
CD247	1q24.2	0,619170096	1,51E-44	8,68E-43
ICOS	2q33.2	0,619082115	1,56E-44	8,97E-43
SLA2	20q11.23	0,619065498	1,57E-44	9,00E-43
ST6GAL1	3q27.3	0,617964564	2,47E-44	1,41E-42
CD3G	11q23.3	0,617832434	2,61E-44	1,48E-42
PTPRJ	11p11.2	0,617577621	2,89E-44	1,64E-42
OSCAR	19q13.42	0,617258879	3,29E-44	1,86E-42
TLR1	4p14	0,6170935	3,52E-44	1,98E-42
CDH11	16q21	0,616909923	3,79E-44	2,13E-42
UBASH3A	21q22.3	0,616474818	4,52E-44	2,54E-42
TNFRSF9	1p36.23	0,616232247	4,99E-44	2,79E-42
NTRK1	1q23.1	0,615926867	5,65E-44	3,15E-42
HEG1	3q21.2	0,615886378	5,74E-44	3,19E-42
ABI3BP	3q12.2	0,615325222	7,20E-44	3,99E-42
MOXD1	6q23.2	0,615211082	7,54E-44	4,17E-42
EBI3	19p13.3	0,614095945	1,18E-43	6,51E-42
GIMAP6	7q36.1	0,6140082	1,22E-43	6,73E-42
LINC00426	13q12.3	0,613993159	1,23E-43	6,75E-42
TNFRSF8	1p36.22	0,613596996	1,44E-43	7,89E-42
TMEM200A	6q23.1	0,613563596	1,46E-43	7,98E-42
FLT3	13q12.2	0,613128578	1,74E-43	9,47E-42
SCML4	6q21	0,613105657	1,76E-43	9,53E-42
JAML	11q23.3	0,612911684	1,90E-43	1,03E-41
PRUNE2	9q21.2	0,612749241	2,03E-43	1,09E-41
C4A	6p21.33	0,612686939	2,08E-43	1,12E-41
PDGFRA	4q12	0,61261167	2,14E-43	1,15E-41
FGFR1	8p11.23	0,611976129	2,76E-43	1,48E-41
AXL	19q13.2	0,611862166	2,89E-43	1,54E-41
GPR174	Xq21.1	0,611064594	3,97E-43	2,11E-41
STK32B	4p16.2	0,610718705	4,55E-43	2,42E-41
KCTD12	13q22.3	0,610587373	4,80E-43	2,54E-41
CYSLTR2	13q14.2	0,610530116	4,91E-43	2,59E-41

CCL23	17q12	0,610019026	6,01E-43	3,16E-41
FYN	6q21	0,609690363	6,84E-43	3,59E-41
TOX	8q12.1	0,609597514	7,10E-43	3,72E-41
ACAP1	17p13.1	0,609509232	7,35E-43	3,84E-41
SYNE3	14q32.13	0,609485026	7,42E-43	3,87E-41
SIGLEC8	19q13.33-q13.41	0,609475803	7,45E-43	3,87E-41
GLT8D2	12q23.3	0,60934509	7,84E-43	4,07E-41
TCF4	18q21.2	0,608800012	9,73E-43	5,03E-41
PAPSS2	10q23.2-q23.31	0,60872068	1,00E-42	5,18E-41
KCNAB2	1p36.31	0,608660077	1,03E-42	5,29E-41
RNASE2	14q11.2	0,608345916	1,16E-42	5,97E-41
GAPT	5q11.2	0,608239334	1,21E-42	6,21E-41
MAFB	20q12	0,607846082	1,42E-42	7,23E-41
LGI2	4p15.2	0,607757032	1,47E-42	7,46E-41
CXCR6	3p21.31	0,607754735	1,47E-42	7,46E-41
CD1D	1q23.1	0,607742544	1,48E-42	7,47E-41
ITGB3	17q21.32	0,607546245	1,59E-42	8,05E-41
CCL2	17q12	0,607307542	1,75E-42	8,82E-41
PALM2AKAP2	9q31.3	0,607213616	1,82E-42	9,13E-41
CCN4	8q24.22	0,607110298	1,89E-42	9,48E-41
VENTX	10q26.3	0,606901497	2,05E-42	1,03E-40
BCL2A1	15q25.1	0,606825455	2,11E-42	1,05E-40
AOX1	2q33.1	0,606736275	2,19E-42	1,09E-40
ANKRD29	18q11.2	0,60666998	2,25E-42	1,12E-40
DNAJC5B	8q13.1	0,606649113	2,26E-42	1,12E-40
GFPT2	5q35.3	0,606240708	2,66E-42	1,31E-40
SAMHD1	20q11.23	0,606067202	2,84E-42	1,40E-40
LAPTM5	1p35.2	0,606039109	2,87E-42	1,41E-40
JAM2	21q21.3	0,605576544	3,44E-42	1,69E-40
DOK3	5q35.3	0,60538961	3,70E-42	1,81E-40
IFI30	19p13.11	0,604971393	4,36E-42	2,13E-40
OLFML2B	1q23.3	0,604828453	4,61E-42	2,24E-40
FAM124A	13q14.3	0,604810276	4,64E-42	2,25E-40
CD22	19q13.12	0,604252739	5,76E-42	2,79E-40
MYLK	3q21.1	0,603929649	6,53E-42	3,16E-40
TSPAN4	11p15.5	0,60382028	6,81E-42	3,29E-40
TIMP2	17q25.3	0,603594651	7,44E-42	3,58E-40
LILRA5	19q13.42	0,603576219	7,49E-42	3,59E-40
DYSF	2p13.2	0,60343475	7,91E-42	3,79E-40
PTGER2	14q22.1	0,603239008	8,53E-42	4,08E-40
CSF1	1p13.3	0,603020774	9,28E-42	4,42E-40
TBC1D10C	11q13.2	0,602846561	9,93E-42	4,72E-40
IL2RB	22q12.3	0,6028114	1,01E-41	4,77E-40
PDCD1	2q37.3	0,602728059	1,04E-41	4,92E-40
ANKRD44	2q33.1	0,60242678	1,17E-41	5,51E-40
LGALS2	22q13.1	0,602201594	1,27E-41	6,00E-40
PLA2G2D	1p36.12	0,602021403	1,37E-41	6,41E-40
JAZF1	7p15.2-p15.1	0,601816302	1,48E-41	6,92E-40
DCLK1	13q13.3	0,601780485	1,50E-41	7,00E-40
LTA	6p21.33	0,601313341	1,79E-41	8,36E-40
CLIC2	Xq28	0,601039765	1,99E-41	9,26E-40
EBF1	5q33.3	0,600952835	2,06E-41	9,56E-40
DPYSL2	8p21.2	0,600724733	2,25E-41	1,04E-39
HLA-DQA2	6p21.32	0,600621018	2,34E-41	1,08E-39
CLECL1	12p13.31	0,600605183	2,35E-41	1,08E-39

P2RX1	17p13.2	0,600527816	2,42E-41	1,11E-39
HS3ST3B1	17p12	0,600305245	2,64E-41	1,21E-39
CD8A	2p11.2	0,600123115	2,83E-41	1,29E-39
TESPA1	12q13.2	0,600033632	2,93E-41	1,34E-39
COL14A1	8q24.12	0,59989996	3,08E-41	1,40E-39
SLC8A1	2p22.1	0,599747656	3,26E-41	1,48E-39
LIX1L	1q21.1	0,599409655	3,71E-41	1,69E-39
MSRB3	12q14.3	0,599278907	3,90E-41	1,77E-39
TRAF3IP3	1q32.2	0,599211086	4,01E-41	1,81E-39
EMILIN1	2p23.3	0,599146215	4,11E-41	1,85E-39
CALD1	7q33	0,598702026	4,86E-41	2,19E-39
RAB3IL1	11q12.2-q12.3	0,598407692	5,44E-41	2,44E-39
ALDH1L2	12q23.3	0,598087978	6,14E-41	2,75E-39
APOE	19q13.32	0,598054293	6,22E-41	2,78E-39
FPR2	19q13.41	0,598028506	6,28E-41	2,80E-39
CRTAM	11q24.1	0,597962957	6,44E-41	2,86E-39
MIR100HG	11q24.1	0,597733607	7,03E-41	3,12E-39
LMCD1	3p25.3	0,597647031	7,26E-41	3,21E-39
PTGDR	14q22.1	0,597528313	7,59E-41	3,35E-39
RHOJ	14q23.2	0,597451439	7,82E-41	3,45E-39
PRRX1	1q24.2	0,59737829	8,04E-41	3,54E-39
FBLN5	14q32.12	0,597306026	8,26E-41	3,63E-39
NAPSB	19q13.33	0,597264001	8,39E-41	3,68E-39
CD79B	17q23.3	0,597123685	8,85E-41	3,87E-39
CLMP	11q24.1	0,597044847	9,12E-41	3,98E-39
SIRPB2	20p13	0,596895936	9,65E-41	4,20E-39
ZAP70	2q11.2	0,596843133	9,84E-41	4,27E-39
CMAHP	6p22.3	0,596577547	1,09E-40	4,71E-39
IL2RG	Xq13.1	0,596376655	1,17E-40	5,07E-39
TBX21	17q21.32	0,596182166	1,26E-40	5,45E-39
ST8SIA4	5q21.1	0,596019951	1,34E-40	5,78E-39
TMEM106A	17q21.31	0,595923807	1,39E-40	5,98E-39
LRRC2	3p21.31	0,5958542	1,43E-40	6,13E-39
CTSK	1q21.3	0,595584216	1,58E-40	6,77E-39
TGFB3	14q24.3	0,595342862	1,73E-40	7,39E-39
MEI1	22q13.2	0,595290739	1,77E-40	7,52E-39
CD72	9p13.3	0,595016875	1,96E-40	8,32E-39
CCL8	17q12	0,594970845	1,99E-40	8,45E-39
FSTL1	3q13.33	0,594838069	2,09E-40	8,86E-39
DCN	12q21.33	0,594159769	2,70E-40	1,14E-38
FMOD	1q32.1	0,593246123	3,80E-40	1,60E-38
SIGLEC11	19q13.33	0,593243556	3,81E-40	1,60E-38
ADAMTS12	5p13.3-p13.2	0,593226176	3,83E-40	1,61E-38
PLA2G4C	19q13.33	0,593131392	3,97E-40	1,66E-38
HSPA12B	20p13	0,593014487	4,15E-40	1,73E-38
FAIM2	12q13.12	0,592998323	4,17E-40	1,74E-38
RASGRP2	11q13.1	0,591564421	7,11E-40	2,96E-38
LRRC32	11q13.5	0,590735055	9,67E-40	4,01E-38
TMEM255A	Xq24	0,590612634	1,01E-39	4,19E-38
CRYBB1	22q12.1	0,590573578	1,03E-39	4,24E-38
COL8A1	3q12.1	0,590317721	1,13E-39	4,65E-38
CPED1	7q31.31	0,590249344	1,16E-39	4,76E-38
RASGRF2	5q14.1	0,590142095	1,20E-39	4,95E-38
CCL4	17q12	0,590011285	1,26E-39	5,18E-38
CRISPLD2	16q24.1	0,589672992	1,43E-39	5,86E-38

HLA-DRB1	6p21.32	0,589644369	1,45E-39	5,91E-38
HIC1	17p13.3	0,589248945	1,67E-39	6,82E-38
CYBRD1	2q31.1	0,589219968	1,69E-39	6,88E-38
PDE1A	2q32.1	0,589201795	1,70E-39	6,91E-38
GZMM	19p13.3	0,589136791	1,74E-39	7,06E-38
TIFAB	5q31.1	0,589055881	1,80E-39	7,26E-38
FAM20C	7p22.3	0,588813412	1,96E-39	7,92E-38
TMEM26	10q21.2	0,588681454	2,06E-39	8,30E-38
SEPTIN6	Xq24	0,588466753	2,23E-39	8,96E-38
TEK	9p21.2	0,588377173	2,31E-39	9,25E-38
FILIP1L	3q12.1	0,588180521	2,48E-39	9,92E-38
ADAM12	10q26.2	0,588070269	2,58E-39	1,03E-37
RUNX3	1p36.11	0,587752057	2,90E-39	1,16E-37
KIAA1755	20q11.23	0,587588131	3,08E-39	1,22E-37
SFMBT2	10p14	0,587352153	3,36E-39	1,33E-37
C11ORF21	11p15.5	0,587241299	3,50E-39	1,38E-37
GLIPR1	12q21.2	0,587154503	3,61E-39	1,43E-37
DCSTAMP	8q22.3	0,587051543	3,75E-39	1,48E-37
ICAM1	19p13.2	0,587038534	3,76E-39	1,48E-37
PEAK3	19p13.3	0,586467107	4,64E-39	1,82E-37
LINC02908	9q34.3	0,586354516	4,83E-39	1,90E-37
MEOX2	7p21.2	0,586317197	4,90E-39	1,92E-37
FUT7	9q34.3	0,585956609	5,58E-39	2,18E-37
SSTR2	17q25.1	0,585908296	5,68E-39	2,22E-37
CXCL9	4q21.1	0,585780765	5,95E-39	2,32E-37
RUNX1T1	8q21.3	0,58569205	6,15E-39	2,39E-37
SPOCK2	10q22.1	0,585195403	7,36E-39	2,86E-37
GUCY1A1	4q32.1	0,58486818	8,29E-39	3,21E-37
ANK2	4q25-q26	0,584609437	9,11E-39	3,52E-37
KLRD1	12p13.2	0,584082258	1,10E-38	4,25E-37
HLA-DRB6	6p21.32	0,583904531	1,18E-38	4,52E-37
SLC2A5	1p36.23	0,583770603	1,23E-38	4,74E-37
COL1A2	7q21.3	0,583632611	1,30E-38	4,97E-37
APBB1IP	10p12.1	0,583492675	1,36E-38	5,22E-37
ADGRA2	8p11.23	0,583467409	1,38E-38	5,26E-37
C1ORF54	1q21.2	0,583377652	1,42E-38	5,42E-37
TNFAIP6	2q23.3	0,583086024	1,58E-38	6,01E-37
ITGA1	5q11.2	0,582577969	1,90E-38	7,20E-37
EPB41L3	18p11.31	0,582554116	1,91E-38	7,25E-37
CTLA4	2q33.2	0,582449091	1,99E-38	7,51E-37
SPARC	5q33.1	0,582411707	2,01E-38	7,60E-37
DIXDC1	11q23.1	0,582393685	2,03E-38	7,64E-37
RGL1	1q25.3	0,58235075	2,06E-38	7,74E-37
KCNJ8	12p12.1	0,582225126	2,15E-38	8,08E-37
ROR2	9q22.31	0,581791361	2,52E-38	9,43E-37
PTGFR	1p31.1	0,581422704	2,87E-38	1,07E-36
GBP5	1p22.2	0,581420849	2,87E-38	1,07E-36
TGM2	20q11.23	0,581416432	2,88E-38	1,07E-36
MSC	8q13.3	0,581294809	3,01E-38	1,12E-36
CPA3	3q24	0,58121477	3,09E-38	1,15E-36
ACSM5	16p12.3	0,581206886	3,10E-38	1,15E-36
COL6A2	21q22.3	0,581079314	3,25E-38	1,20E-36
BATF3	1q32.3	0,581028012	3,31E-38	1,22E-36
FMO2	1q24.3	0,580921996	3,43E-38	1,27E-36
EDNRA	4q31.22-q31.23	0,580747674	3,66E-38	1,34E-36

ACVRL1	12q13.13	0,580707036	3,71E-38	1,36E-36
SULF1	8q13.2-q13.3	0,580634241	3,81E-38	1,40E-36
COL3A1	2q32.2	0,580479287	4,02E-38	1,47E-36
SIRPA	20p13	0,580228569	4,40E-38	1,61E-36
CCL19	9p13.3	0,580207174	4,43E-38	1,62E-36
KCNE4	2q36.1	0,580177683	4,48E-38	1,63E-36
ADAMTS4	1q23.3	0,58012185	4,57E-38	1,66E-36
EOMES	3p24.1	0,579728216	5,26E-38	1,91E-36
FGF7	15q21.2	0,57961476	5,48E-38	1,98E-36
FYB1	5p13.1	0,579430299	5,85E-38	2,11E-36
CD200	3q13.2	0,578537326	8,03E-38	2,90E-36
CADM3	1q23.2	0,578462511	8,25E-38	2,97E-36
NEFH	22q12.2	0,578085401	9,43E-38	3,39E-36
LEF1	4q25	0,577612018	1,11E-37	4,00E-36
DENND5A	11p15.4	0,577476676	1,17E-37	4,19E-36
SH3RF3	2q13	0,577462718	1,18E-37	4,20E-36
CD6	11q12.2	0,577268893	1,26E-37	4,49E-36
PIPOX	17q11.2	0,577024602	1,37E-37	4,88E-36
GPR132	14q32.33	0,576925591	1,42E-37	5,05E-36
SIRPB1	20p13	0,576899909	1,43E-37	5,09E-36
SDS	12q24.13	0,576487409	1,66E-37	5,87E-36
CEACAM4	19q13.2	0,576471718	1,67E-37	5,89E-36
SLC2A3	12p13.31	0,576421151	1,70E-37	5,99E-36
SNED1	2q37.3	0,576045516	1,94E-37	6,82E-36
GUCY1B1	4q32.1	0,575682425	2,20E-37	7,74E-36
ABCC9	12p12.1	0,575426936	2,41E-37	8,45E-36
PRF1	10q22.1	0,575147418	2,66E-37	9,30E-36
LOXL3	2p13.1	0,575122682	2,68E-37	9,37E-36
PDLIM3	4q35.1	0,575111021	2,69E-37	9,39E-36
F2RL2	5q13.3	0,574818052	2,98E-37	1,04E-35
CPXM1	20p13	0,574681849	3,13E-37	1,09E-35
TLN1	9p13.3	0,574189071	3,71E-37	1,29E-35
FILIP1	6q14.1	0,574071474	3,87E-37	1,34E-35
CCN1	1p22.3	0,574019098	3,94E-37	1,36E-35
FNBP1	9q34.11	0,573979167	4,00E-37	1,38E-35
ARSB	5q14.1	0,573829867	4,21E-37	1,45E-35
VNN2	6q23.2	0,573439138	4,82E-37	1,66E-35
HLA-DQB1	6p21.32	0,573260583	5,13E-37	1,77E-35
FBXL7	5p15.1	0,5730816	5,46E-37	1,88E-35
EMP3	19q13.33	0,573040962	5,54E-37	1,90E-35
ADORA2A	22q11.23	0,572707201	6,22E-37	2,13E-35
FAM124B	2q36.2	0,57270031	6,24E-37	2,13E-35
AEBP1	7p13	0,572501538	6,69E-37	2,28E-35
TSPAN32	11p15.5	0,571972239	8,03E-37	2,73E-35
CREB5	7p15.1-p14.3	0,57183278	8,43E-37	2,87E-35
LUM	12q21.33	0,571283461	1,02E-36	3,46E-35
LCK	1p35.2	0,571071438	1,10E-36	3,72E-35
SERPING1	11q12.1	0,571027973	1,11E-36	3,77E-35
MARCHF1	4q32.2-q32.3	0,570412574	1,38E-36	4,65E-35
CCN2	6q23.2	0,570256029	1,45E-36	4,90E-35
AKT3	1q43-q44	0,570201963	1,48E-36	4,98E-35
SH2D3C	9q34.11	0,570171333	1,50E-36	5,03E-35
RASL12	15q22.31	0,569810779	1,70E-36	5,68E-35
HEPH	Xq12	0,569764841	1,72E-36	5,77E-35
CYP27A1	2q35	0,569694166	1,77E-36	5,90E-35

CYRIA	2p24.2	0,569513593	1,88E-36	6,26E-35
PDZRN3	3p13	0,569303336	2,02E-36	6,72E-35
ADAM19	5q33.3	0,5691922	2,10E-36	6,97E-35
TNFRSF4	1p36.33	0,569166227	2,12E-36	7,02E-35
CSGALNACT2	10q11.21	0,569060745	2,19E-36	7,27E-35
POU2F2	19q13.2	0,568857202	2,35E-36	7,78E-35
DCANP1	5q31.1	0,56881544	2,39E-36	7,88E-35
LHFPL6	13q13.3-q14.11	0,568255231	2,89E-36	9,54E-35
KCNMB1	5q35.1	0,568141735	3,01E-36	9,88E-35
SHE	1q21.3	0,568141269	3,01E-36	9,88E-35
DOK5	20q13.2	0,567928216	3,24E-36	1,06E-34
VASH1	14q24.3	0,567586826	3,64E-36	1,19E-34
PKDCC	2p21	0,567522714	3,72E-36	1,22E-34
GXYLT2	3p13	0,567508374	3,74E-36	1,22E-34
POSTN	13q13.3	0,567289993	4,02E-36	1,31E-34
CD40LG	Xq26.3	0,566830312	4,71E-36	1,53E-34
PTGIR	19q13.32	0,566798565	4,76E-36	1,55E-34
VCAN	5q14.2-q14.3	0,566202488	5,83E-36	1,89E-34
MAP1B	5q13.2	0,566182699	5,87E-36	1,90E-34
A2M	12p13.31	0,565966081	6,32E-36	2,04E-34
FERMT2	14q22.1	0,56559716	7,16E-36	2,31E-34
ADAMTS10	19p13.2	0,565579163	7,20E-36	2,32E-34
NKG7	19q13.41	0,565490378	7,42E-36	2,39E-34
ARHGAP20	11q22.3-q23.1	0,565189657	8,22E-36	2,64E-34
SCN1B	19q13.11	0,565001373	8,76E-36	2,81E-34
HRH2	5q35.2	0,564949136	8,92E-36	2,86E-34
GPR176	15q14-q15.1	0,564890237	9,10E-36	2,91E-34
GRK5	10q26.11	0,564567784	1,01E-35	3,24E-34
MRO	18q21.2	0,564542519	1,02E-35	3,26E-34
LAMA4	6q21	0,564306818	1,11E-35	3,53E-34
CLIC4	1p36.11	0,564037901	1,21E-35	3,86E-34
BOC	3q13.2	0,563894609	1,27E-35	4,04E-34
FAP	2q24.2	0,563836479	1,30E-35	4,11E-34
RGS2	1q31.2	0,563752553	1,34E-35	4,22E-34
RFTN1	3p24.3	0,563616504	1,40E-35	4,42E-34
KLRG1	12p13.31	0,56351364	1,45E-35	4,56E-34
MIR155HG	21q21.3	0,56342197	1,49E-35	4,70E-34
NTNG2	9q34.13	0,563308009	1,55E-35	4,88E-34
CORO1A	16p11.2	0,563293875	1,56E-35	4,89E-34
SHISAL1	22q13.31	0,563265251	1,57E-35	4,93E-34
TNFAIP8L3	15q21.2	0,563222341	1,60E-35	5,00E-34
SARDH	9q34.2	0,563123372	1,65E-35	5,16E-34
FNDC1	6q25.3	0,563072819	1,68E-35	5,24E-34
CEBPE	14q11.2	0,562822011	1,83E-35	5,69E-34
ZNF469	16q24.2	0,56280675	1,84E-35	5,71E-34
PLCB2	15q15.1	0,56251398	2,03E-35	6,29E-34
UNC5A	5q35.2	0,562361981	2,13E-35	6,61E-34
PLA1A	3q13.33	0,562278846	2,19E-35	6,79E-34
MRAS	3q22.3	0,561910419	2,48E-35	7,67E-34
GPBAR1	2q35	0,561802994	2,57E-35	7,94E-34
CCR6	6q27	0,561742214	2,63E-35	8,09E-34
SLC2A14	12p13.31	0,56166073	2,70E-35	8,30E-34
PCDHGA12	5q31.3	0,561566399	2,79E-35	8,55E-34
CD74	5q33.1	0,56138301	2,96E-35	9,08E-34
CLEC7A	12p13.2	0,56097133	3,40E-35	1,04E-33

KLRB1	12p13.31	0,560935229	3,44E-35	1,05E-33
CXCR5	11q23.3	0,560781063	3,62E-35	1,11E-33
FCRL5	1q23.1	0,560680908	3,75E-35	1,14E-33
DCHS1	11p15.4	0,560480319	4,00E-35	1,22E-33
SYNPO	5q33.1	0,560465477	4,02E-35	1,22E-33
ZFPM2	8q23	0,560225007	4,36E-35	1,32E-33
RAB39A	11q22.3	0,559666687	5,25E-35	1,59E-33
XPNPEP2	Xq26.1	0,55960442	5,36E-35	1,62E-33
VSTM4	10q11.23	0,559487694	5,57E-35	1,68E-33
MAMLD1	Xq28	0,559427974	5,69E-35	1,71E-33
RASGRP1	15q14	0,559378261	5,78E-35	1,74E-33
SIGLEC17P	19q13.41	0,558790137	7,03E-35	2,11E-33
CFP	Xp11.23	0,558473891	7,80E-35	2,34E-33
IGSF21	1p36.13	0,558098488	8,84E-35	2,65E-33
FGF2	4q28.1	0,558079149	8,89E-35	2,66E-33
KLF9	9q21.12	0,557895572	9,45E-35	2,82E-33
KLRK1	12p13.2	0,557788323	9,79E-35	2,92E-33
XIRP1	3p22.2	0,557594512	1,04E-34	3,11E-33
BACH2	6q15	0,557380001	1,12E-34	3,33E-33
CCL18	17q12	0,557230489	1,18E-34	3,49E-33
SP140	2q37.1	0,55713905	1,21E-34	3,60E-33
STARD13	13q13.1	0,556694482	1,40E-34	4,16E-33
BIN1	2q14.3	0,556523425	1,49E-34	4,39E-33
LTB	6p21.33	0,556427508	1,53E-34	4,53E-33
DPP4	2q24.2	0,55615309	1,68E-34	4,95E-33
NNMT	11q23.2	0,556024109	1,75E-34	5,15E-33
P4HA3	11q13.4	0,556021635	1,75E-34	5,15E-33
CLEC6A	12p13.31	0,555981698	1,78E-34	5,21E-33
RASGEF1B	4q21.21	0,555926224	1,81E-34	5,30E-33
PTGDS	9q34.3	0,555288916	2,23E-34	6,52E-33
MEDAG	13q12.3	0,555251812	2,26E-34	6,59E-33
JCAD	10p11.23	0,555121947	2,35E-34	6,87E-33
SPARCL1	4q22.1	0,55500074	2,45E-34	7,13E-33
RASA3	13q34	0,554771225	2,64E-34	7,68E-33
CD7	17q25.3	0,554338696	3,04E-34	8,83E-33
STAP1	4q13.2	0,554286766	3,09E-34	8,97E-33
TNS1	2q35	0,554189926	3,19E-34	9,25E-33
ADGRG5	16q21	0,554050098	3,34E-34	9,66E-33
ANPEP	15q26.1	0,553797682	3,63E-34	1,05E-32
CCDC102B	18q22.1	0,553725417	3,71E-34	1,07E-32
ADGRE4P	19p13.2	0,553511046	3,98E-34	1,15E-32
PLXND1	3q22.1	0,553498905	4,00E-34	1,15E-32
ANKRD55	5q11.2	0,553148565	4,48E-34	1,29E-32
COL8A2	1p34.3	0,552952237	4,78E-34	1,37E-32
PXDN	2p25.3	0,55288545	4,88E-34	1,40E-32
TLR10	4p14	0,552822116	4,98E-34	1,43E-32
PTGS1	9q33.2	0,552723428	5,15E-34	1,47E-32
EPB41L2	6q23.1-q23.2	0,552653637	5,27E-34	1,50E-32
ITGA11	15q23	0,552562957	5,42E-34	1,54E-32
ADAMTS6	5q12.3	0,552370506	5,77E-34	1,64E-32
PECAM1	17q23.3	0,552141423	6,22E-34	1,76E-32
CD3D	11q23.3	0,552120495	6,26E-34	1,77E-32
IGDCC4	15q22.31	0,551931519	6,66E-34	1,88E-32
CD79A	19q13.2	0,551808128	6,93E-34	1,96E-32
FXYD6	11q23.3	0,551715255	7,14E-34	2,01E-32

PIK3CD	1p36.22	0,551633626	7,33E-34	2,06E-32
MIAT	22q12.1	0,551630445	7,34E-34	2,06E-32
OSM	22q12.2	0,551398553	7,91E-34	2,22E-32
FCRL2	1q23.1	0,551345379	8,05E-34	2,26E-32
ANGPTL1	1q25.2	0,551333181	8,08E-34	2,26E-32
GGT5	22q11.23	0,551208695	8,41E-34	2,35E-32
ZNF804A	2q32.1	0,55117518	8,50E-34	2,38E-32
HPGDS	4q22.3	0,550706762	9,89E-34	2,76E-32
CCL4L1	17q12 alternate reference locus	0,550250325	1,15E-33	3,19E-32
GREM1	15q13.3	0,549781023	1,33E-33	3,71E-32
SV2B	15q26.1	0,54970247	1,37E-33	3,80E-32
ZNF80	3q13.31	0,549359844	1,53E-33	4,23E-32
TMC8	17q25.3	0,549322566	1,54E-33	4,28E-32
LZTS1	8p21.3	0,549315676	1,55E-33	4,28E-32
SLC15A3	11q12.2	0,549213374	1,60E-33	4,42E-32
PLSCR4	3q24	0,549184044	1,61E-33	4,46E-32
CD300E	17q25.1	0,54917641	1,62E-33	4,46E-32
FAM180A	7q33	0,549085717	1,67E-33	4,59E-32
STX11	6q24.2	0,549028207	1,70E-33	4,67E-32
IL3RA	Xp22.3 and Yp13.3	0,548999053	1,71E-33	4,70E-32
CXCL13	4q21.1	0,548941093	1,75E-33	4,78E-32
FCAR	19q13.42	0,548847368	1,80E-33	4,92E-32
SGCD	5q33.2-q33.3	0,548569076	1,97E-33	5,38E-32
UNC5C	4q22.3	0,548080101	2,30E-33	6,28E-32
CPXM2	10q26.13	0,547986993	2,37E-33	6,46E-32
TNFRSF13B	17p11.2	0,547480969	2,78E-33	7,58E-32
MMD	17q22	0,547299153	2,95E-33	8,02E-32
PRKG1	10q11.23-q21.1	0,54729651	2,95E-33	8,02E-32
PRICKLE1	12q12	0,547241553	3,01E-33	8,15E-32
COL1A1	17q21.33	0,547136778	3,11E-33	8,42E-32
COL5A1	9q34.3	0,546662374	3,62E-33	9,78E-32
FMO1	1q24.3	0,546537681	3,76E-33	1,02E-31
MAP1A	15q15.3	0,546402822	3,93E-33	1,06E-31
CCL3	17q12	0,546240471	4,13E-33	1,11E-31
TMEM119	12q23.3	0,546173483	4,22E-33	1,14E-31
NAV3	12q21.2	0,546127618	4,29E-33	1,15E-31
DENND1C	19p13.3	0,545915344	4,58E-33	1,23E-31
CILP	15q22.31	0,545636806	5,01E-33	1,34E-31
CCL5	17q12	0,545601549	5,06E-33	1,35E-31
ALPK2	18q21.31-q21.32	0,545576949	5,10E-33	1,36E-31
ST3GAL6	3q12.1	0,545573632	5,11E-33	1,36E-31
RGS13	1q31.2	0,545477901	5,27E-33	1,40E-31
GNB4	3q26.33	0,545431046	5,35E-33	1,42E-31
LAMA2	6q22.33	0,545382281	5,43E-33	1,44E-31
CHI3L1	1q32.1	0,545349353	5,49E-33	1,46E-31
COL5A2	2q32.2	0,54469285	6,75E-33	1,79E-31
SPON1	11p15.2	0,544683662	6,77E-33	1,79E-31
TNIP3	4q27	0,544609409	6,93E-33	1,83E-31
LOX	5q23.1	0,544279404	7,70E-33	2,03E-31
C1QTNF1	17q25.3	0,544263502	7,73E-33	2,04E-31
JCHAIN	4q13.3	0,544223747	7,83E-33	2,06E-31
AQP9	15q21.3	0,544064981	8,24E-33	2,17E-31
P2RY12	3q25.1	0,543887358	8,71E-33	2,29E-31
MAPRE2	18q12.1-q12.2	0,543869668	8,76E-33	2,30E-31

TRIL	7p14.3	0,543818011	8,90E-33	2,33E-31
CTHRC1	8q22.3	0,543816308	8,91E-33	2,33E-31
NRXN2	11q13.1	0,543783268	9,00E-33	2,35E-31
HLA-DRB5	6p21.32	0,543457281	9,97E-33	2,60E-31
IL4I1	19q13.33	0,543415053	1,01E-32	2,63E-31
KIF19	17q25.1	0,543395948	1,02E-32	2,65E-31
TIE1	1p34.2	0,543201969	1,08E-32	2,81E-31
DACT1	14q23.1	0,542848773	1,21E-32	3,13E-31
SERPINF1	17p13.3	0,542598762	1,31E-32	3,39E-31
COL6A1	21q22.3	0,542208461	1,48E-32	3,82E-31
IL18RAP	2q12.1	0,542177313	1,49E-32	3,86E-31
TPP1	11p15.4	0,542169943	1,49E-32	3,86E-31
SLCO5A1	8q13.3	0,542034799	1,56E-32	4,02E-31
GAS1	9q21.33	0,541971701	1,59E-32	4,10E-31
JAM3	11q25	0,54193389	1,61E-32	4,14E-31
SLFN11	17q12	0,541812683	1,67E-32	4,29E-31
SLC43A3	11q12.1	0,541491291	1,85E-32	4,74E-31
GLI2	2q14.2	0,541368365	1,92E-32	4,92E-31
CREM	10p11.21	0,541229971	2,01E-32	5,13E-31
RASGRP3	2p22.3	0,541160822	2,05E-32	5,24E-31
SIRPG	20p13	0,540709341	2,36E-32	6,02E-31
GZMA	5q11.2	0,54066255	2,40E-32	6,11E-31
FIBIN	11p14.2	0,540483148	2,53E-32	6,45E-31
CHST13	3q21.3	0,540404605	2,60E-32	6,60E-31
CRHBP	5q13.3	0,540240076	2,73E-32	6,94E-31
KLHL4	Xq21.31	0,540020346	2,93E-32	7,42E-31
SFRP4	7p14.1	0,539839924	3,09E-32	7,84E-31
GPR18	13q32.3	0,539696456	3,24E-32	8,19E-31
PLXDC1	17q12	0,539613113	3,32E-32	8,39E-31
SGIP1	1p31.3	0,539474238	3,47E-32	8,75E-31
PTPRD	9p24.1-p23	0,539023341	3,99E-32	1,01E-30
EPHA3	3p11.1	0,539019446	3,99E-32	1,01E-30
BHLHE22	8q12.3	0,538906752	4,14E-32	1,04E-30
GPR84	12q13.13	0,538890489	4,16E-32	1,04E-30
SPIB	19q13.33	0,538788546	4,29E-32	1,08E-30
CALCRL	2q32.1	0,53872491	4,38E-32	1,10E-30
TREML2	6p21.1	0,538674519	4,44E-32	1,11E-30
TRAM2	6p12.2	0,538543276	4,63E-32	1,16E-30
MZB1	5q31.2	0,538515142	4,67E-32	1,17E-30
PNOC	8p21.1	0,538464898	4,74E-32	1,18E-30
THY1	11q23.3	0,538411292	4,82E-32	1,20E-30
GLT1D1	12q24.33	0,538242516	5,08E-32	1,26E-30
PRR16	5q23.1	0,538236019	5,09E-32	1,26E-30
ARID5A	2q11.2	0,538231955	5,10E-32	1,26E-30
SSC5D	19q13.42	0,538222702	5,11E-32	1,27E-30
GBGT1	9q34.2	0,538124706	5,27E-32	1,30E-30
GRIP2	3p25.1	0,538076455	5,35E-32	1,32E-30
RENBP	Xq28	0,538059444	5,38E-32	1,33E-30
CLEC9A	12p13.2	0,537822948	5,78E-32	1,43E-30
GASK1B	4q32.1	0,537633871	6,13E-32	1,51E-30
DAAM2	6p21.2	0,537576978	6,24E-32	1,53E-30
JAK3	19p13.11	0,537407536	6,58E-32	1,61E-30
GAL3ST4	7q22.1	0,537115473	7,20E-32	1,76E-30
DPT	1q24.2	0,53692624	7,63E-32	1,87E-30
PODN	1p32.3	0,536925358	7,63E-32	1,87E-30

MS4A1	11q12.2	0,536862956	7,78E-32	1,90E-30
SFRP2	4q31.3	0,536856034	7,80E-32	1,90E-30
CSMD2	1p35.1	0,536525364	8,63E-32	2,10E-30
NCR3	6p21.33	0,536431008	8,89E-32	2,16E-30
FN1	2q35	0,536252536	9,39E-32	2,28E-30
CXCR2P1	2q35	0,536180202	9,60E-32	2,33E-30
MILR1	17q23.3	0,53582212	1,07E-31	2,60E-30
STAT4	2q32.2-q32.3	0,535610923	1,14E-31	2,77E-30
GPX8	5q11.2	0,535457093	1,20E-31	2,90E-30
ST8SIA1	12p12.1	0,535369039	1,23E-31	2,98E-30
BMS1P20	22q11.22	0,535007956	1,38E-31	3,32E-30
NEGR1	1p31.1	0,535003716	1,38E-31	3,32E-30
STX2	12q24.33	0,534994705	1,38E-31	3,32E-30
SVEP1	9q31.3	0,534874205	1,43E-31	3,45E-30
ZCCHC24	10q22.3	0,534850705	1,44E-31	3,47E-30
PIK3CD-AS1	1p36.22	0,534503388	1,60E-31	3,85E-30
AKAP5	14q23.3	0,53445928	1,63E-31	3,90E-30
CCL13	17q12	0,534332803	1,69E-31	4,05E-30
ISLR	15q24.1	0,534304391	1,71E-31	4,08E-30
CSGALNACT1	8p21.3	0,533801011	1,99E-31	4,75E-30
CPEB1	15q25.2	0,533690217	2,06E-31	4,91E-30
SMAP2	1p34.2	0,533089317	2,47E-31	5,88E-30
CCND2	12p13.32	0,533076773	2,48E-31	5,90E-30
ZNF366	5q13.1	0,533005834	2,53E-31	6,02E-30
IRAG1	11p15.4	0,532814217	2,69E-31	6,37E-30
IL27	16p12.1-p11.2	0,532719884	2,76E-31	6,55E-30
ASGR2	17p13.1	0,53270394	2,78E-31	6,58E-30
IGFLR1	19q13.12	0,532631346	2,84E-31	6,71E-30
ADGRD1	12q24.33	0,532283482	3,15E-31	7,45E-30
SPATC1	8q24.3	0,532268429	3,17E-31	7,48E-30
CIITA	16p13.13	0,532060407	3,38E-31	7,96E-30
ST3GAL2	16q22.1	0,53195234	3,49E-31	8,21E-30
ETV1	7p21.2	0,531820355	3,63E-31	8,54E-30
GRAP	17p11.2	0,531737312	3,72E-31	8,74E-30
HLA-DOB	6p21.32	0,531645106	3,83E-31	8,98E-30
CALU	7q32.1	0,53150921	3,99E-31	9,35E-30
PTCRA	6p21.1	0,531329486	4,21E-31	9,86E-30
SERPINA1	14q32.13	0,531260789	4,30E-31	1,01E-29
PDGFC	4q32.1	0,530996466	4,66E-31	1,09E-29
PCSK5	9q21.13	0,530647674	5,17E-31	1,21E-29
LAG3	12p13.31	0,530632492	5,20E-31	1,21E-29
PRKCB	16p12.2-p12.1	0,530623858	5,21E-31	1,21E-29
ADAM6	14q32.33	0,530605812	5,24E-31	1,22E-29
SGTB	5q12.3	0,530425062	5,53E-31	1,28E-29
TMEM140	7q33	0,530391315	5,59E-31	1,30E-29
HVCN1	12q24.11	0,530340959	5,68E-31	1,31E-29
IFITM2	11p15.5	0,530264742	5,81E-31	1,34E-29
TCN2	22q12.2	0,529914261	6,45E-31	1,49E-29
APLNR	11q12.1	0,529693933	6,90E-31	1,59E-29
SOD2	6q25.3	0,529516893	7,27E-31	1,68E-29
LRIG1	3p14.1	0,529467598	7,38E-31	1,70E-29
GPR55	2q37.1	0,529270211	7,83E-31	1,80E-29
APOC2	19q13.32	0,528857821	8,87E-31	2,04E-29
PCDH18	4q28.3	0,528668621	9,38E-31	2,15E-29
CAVIN1	17q21.2	0,528508897	9,84E-31	2,26E-29

CD248	11q13.2	0,528122483	1,11E-30	2,53E-29
THBS1	15q14	0,527592954	1,29E-30	2,96E-29
CHN1	2q31.1	0,527431639	1,36E-30	3,10E-29
RNF130	5q35.3	0,526995223	1,55E-30	3,53E-29
MAP3K3	17q23.3	0,526483186	1,80E-30	4,11E-29
PCDHGB7	5q31.3	0,526432026	1,83E-30	4,17E-29
TSPAN11	12p11.21	0,526184586	1,97E-30	4,48E-29
NAP1L3	Xq21.32	0,526172552	1,98E-30	4,49E-29
MFGE8	15q26.1	0,526146952	1,99E-30	4,52E-29
MAP1LC3C	1q43	0,526029398	2,06E-30	4,67E-29
RAB27A	15q21.3	0,525949769	2,11E-30	4,78E-29
SYNE1	6q25.2	0,525581025	2,36E-30	5,33E-29
CYP1B1	2p22.2	0,525526805	2,39E-30	5,41E-29
LCN10	9q34.3	0,525298315	2,56E-30	5,78E-29
RGS4	1q23.3	0,525259279	2,59E-30	5,84E-29
NRP1	10p11.22	0,524960855	2,83E-30	6,37E-29
ARHGAP18	6q22.33	0,524555006	3,19E-30	7,18E-29
CCPG1	15q21.3	0,524284676	3,46E-30	7,77E-29
ACP5	19p13.2	0,524220539	3,53E-30	7,91E-29
PTGER3	1p31.1	0,524094474	3,66E-30	8,20E-29
PTPRCAP	11q13.2	0,523989433	3,77E-30	8,45E-29
COL16A1	1p35.2	0,523959219	3,81E-30	8,51E-29
OMD	9q22.31	0,523937582	3,83E-30	8,56E-29
CD69	12p13.31	0,523874168	3,90E-30	8,71E-29
MYADM	19q13.42	0,523787303	4,01E-30	8,93E-29
CLIC5	6p21.1	0,523516973	4,34E-30	9,66E-29
COL4A2	13q34	0,523439938	4,44E-30	9,87E-29
ZMYND15	17p13.2	0,522924543	5,17E-30	1,15E-28
SLC16A2	Xq13.2	0,52271994	5,49E-30	1,22E-28
CHRDL2	11q13.4	0,522649699	5,60E-30	1,24E-28
TPSAB1	16p13.3	0,522617992	5,65E-30	1,25E-28
LYVE1	11p15.4	0,522591312	5,70E-30	1,26E-28
ZBP1	20q13.31	0,522511574	5,83E-30	1,29E-28
TPSB2	16p13.3	0,522382646	6,06E-30	1,34E-28
PLCL2	3p24.3	0,522349429	6,12E-30	1,35E-28
MRC2	17q23.2	0,522287412	6,23E-30	1,37E-28
ANGPTL2	9q33.3	0,522120973	6,54E-30	1,44E-28
PLXNA4	7q32.3	0,521955381	6,87E-30	1,51E-28
FASLG	1q24.3	0,521869284	7,04E-30	1,54E-28
MN1	22q12.1	0,521708057	7,38E-30	1,62E-28
PIM2	Xp11.23	0,521435959	7,99E-30	1,75E-28
IL6	7p15.3	0,521269598	8,39E-30	1,84E-28
RBMS3	3p24.1	0,521239534	8,47E-30	1,85E-28
GRAMD1B	11q24.1	0,521130013	8,74E-30	1,91E-28
RAPGEF1	9q34.13	0,52105679	8,93E-30	1,95E-28
PLN	6q22.31	0,520899414	9,35E-30	2,04E-28
RBM24	6p22.3	0,520754066	9,76E-30	2,12E-28
PTPRO	12p12.3	0,520669163	1,00E-29	2,17E-28
CLIP3	19q13.12	0,520319477	1,11E-29	2,40E-28
TLR6	4p14	0,520118876	1,17E-29	2,55E-28
GZMH	14q12	0,52004587	1,20E-29	2,60E-28
ARL4C	2q37.1	0,520022998	1,21E-29	2,61E-28
ARHGAP24	4q21.23-q21.3	0,519968159	1,23E-29	2,65E-28
PLPP7	9q34.13	0,51993311	1,24E-29	2,68E-28
ARRB2	17p13.2	0,519307417	1,49E-29	3,21E-28

PLEKHO1	1q21.2	0,519041151	1,61E-29	3,46E-28
LRRC15	3q29	0,518926223	1,66E-29	3,57E-28
DPEP1	16q24.3	0,518924495	1,66E-29	3,57E-28
NR4A3	9q22	0,518771527	1,74E-29	3,73E-28
HAPLN3	15q26.1	0,51877082	1,74E-29	3,73E-28
CFL2	14q13.1	0,518751915	1,75E-29	3,74E-28
RORA	15q22.2	0,518695198	1,78E-29	3,80E-28
KLHL5	4p14	0,518179097	2,06E-29	4,41E-28
STK17B	2q32.3	0,518105242	2,11E-29	4,50E-28
LAYN	11q23.1	0,517891563	2,24E-29	4,78E-28
FCRL1	1q23.1	0,517870958	2,26E-29	4,81E-28
TRAF1	9q33.2	0,517862475	2,26E-29	4,81E-28
VAV1	19p13.3	0,517860885	2,26E-29	4,81E-28
LRFN5	14q21.1	0,517817488	2,29E-29	4,87E-28
CCIN	9p13.3	0,517753555	2,33E-29	4,95E-28
FBLN2	3p25.1	0,517750102	2,34E-29	4,95E-28
SNAI1	20q13.13	0,517658932	2,40E-29	5,08E-28
FAT4	4q28.1	0,517403267	2,58E-29	5,46E-28
SLAMF7	1q23.3	0,517256264	2,69E-29	5,69E-28
BCL6B	17p13.1	0,517192656	2,74E-29	5,79E-28
SNAP25	20p12.2	0,51706215	2,85E-29	6,01E-28
PALLD	4q32.3	0,516930277	2,96E-29	6,23E-28
GPR68	14q32.11	0,516554288	3,30E-29	6,94E-28
SUSD3	9q22.31	0,516400394	3,45E-29	7,25E-28
C2	6p21.33	0,516360816	3,49E-29	7,32E-28
MATK	19p13.3	0,516355804	3,49E-29	7,32E-28
TNFRSF17	16p13.13	0,516068365	3,79E-29	7,95E-28
TOX2	20q13.12	0,515780401	4,12E-29	8,62E-28
MEFV	16p13.3	0,51521089	4,85E-29	1,01E-27
TNFSF14	19p13.3	0,514922197	5,27E-29	1,10E-27
GATA6	18q11.2	0,514705087	5,61E-29	1,17E-27
APBB2	4p14-p13	0,514605083	5,77E-29	1,20E-27
ENPP2	8q24.12	0,514247646	6,39E-29	1,33E-27
IL18R1	2q12.1	0,514178561	6,52E-29	1,36E-27
GALNT17	7q11.22	0,514106014	6,65E-29	1,38E-27
BGN	Xq28	0,514034209	6,79E-29	1,41E-27
FFAR3	19q13.12	0,514003476	6,85E-29	1,42E-27
TGFBR2	3p24.1	0,51390187	7,05E-29	1,46E-27
ADGRL4	1p31.1	0,513769886	7,32E-29	1,52E-27
CD70	19p13.3	0,513747659	7,37E-29	1,52E-27
RGL4	22q11.23	0,513169744	8,69E-29	1,79E-27
CD163L1	12p13.31	0,513112082	8,83E-29	1,82E-27
MS4A14	11q12.2	0,513064399	8,96E-29	1,85E-27
CCL11	17q12	0,512926253	9,31E-29	1,92E-27
PDE3A	12p12.2	0,512690313	9,96E-29	2,05E-27
LATS2	13q12.11	0,512594214	1,02E-28	2,10E-27
TRPS1	8q23.3	0,512465409	1,06E-28	2,18E-27
PPFIA2	12q21.31	0,512353857	1,10E-28	2,25E-27
GALNT15	3p25.1	0,512174407	1,15E-28	2,36E-27
KIF17	1p36.12	0,51205956	1,19E-28	2,44E-27
NCF4	22q12.3	0,511863615	1,26E-28	2,57E-27
FKBP5	6p21.31	0,511774742	1,29E-28	2,64E-27
LPAR4	Xq21.1	0,511748017	1,30E-28	2,65E-27
GAS6	13q34	0,511739758	1,30E-28	2,66E-27
FLNC	7q32.1	0,511592225	1,36E-28	2,77E-27

ADAMTS3	4q13.3	0,511492999	1,40E-28	2,85E-27
HTR2A	13q14.2	0,511455114	1,41E-28	2,87E-27
CACNA2D4	12p13.33	0,511334992	1,46E-28	2,97E-27
POMC	2p23.3	0,511313197	1,47E-28	2,98E-27
EFEMP1	2p16.1	0,511179485	1,53E-28	3,10E-27
REM1	20q11.21	0,511081602	1,57E-28	3,18E-27
CAMK1G	1q32.2	0,510780794	1,71E-28	3,46E-27
SYNM	15q26.3	0,510767982	1,72E-28	3,47E-27
CD19	16p11.2	0,510632476	1,78E-28	3,60E-27
OTOA	16p12.2	0,510603173	1,80E-28	3,63E-27
PDPN	1p36.21	0,510589529	1,81E-28	3,64E-27
ANTXR1	2p13.3	0,510363547	1,93E-28	3,87E-27
ADRA2A	10q25.2	0,510223258	2,00E-28	4,02E-27
GZMB	14q12	0,510173345	2,03E-28	4,08E-27
TLDC2	20q11.23	0,510103665	2,07E-28	4,15E-27
NCAM1	11q23.2	0,510057217	2,10E-28	4,20E-27
CYTL1	4p16.2	0,510036067	2,11E-28	4,23E-27
SCN7A	2q24.3	0,509792832	2,26E-28	4,52E-27
ISLR2	15q24.1	0,509792532	2,26E-28	4,52E-27
ARHGAP30	1q23.3	0,509678356	2,34E-28	4,66E-27
CTSB	8p23.1	0,509445484	2,49E-28	4,97E-27
VMO1	17p13.2	0,509369973	2,55E-28	5,07E-27
STOM	9q33.2	0,509354313	2,56E-28	5,09E-27
TARP	7p14.1	0,509238152	2,64E-28	5,25E-27
PNMA2	8p21.2	0,508986298	2,84E-28	5,63E-27
CSDC2	22q13.2	0,508938087	2,88E-28	5,70E-27
CTSL	9q21.33	0,508828848	2,97E-28	5,87E-27
TRPC3	4q27	0,508752696	3,03E-28	6,00E-27
CFI	4q25	0,508679724	3,09E-28	6,11E-27
PROS1	3q11.1	0,508557634	3,20E-28	6,32E-27
HS3ST3A1	17p12	0,508523436	3,23E-28	6,37E-27
TPM1	15q22.2	0,50852	3,23E-28	6,37E-27
EPSTI1	13q14.11	0,508405684	3,34E-28	6,58E-27
MCHR1	22q13.2	0,50827125	3,47E-28	6,82E-27
SUCNR1	3q25.1	0,508250767	3,49E-28	6,85E-27
SMOC2	6q27	0,508018917	3,72E-28	7,31E-27
ANKRD36BP2	2p11.2	0,507910297	3,84E-28	7,52E-27
NIBAN1	1q25.3	0,507742932	4,02E-28	7,88E-27
KIRREL1	1q23.1	0,507709208	4,06E-28	7,94E-27
APCDD1L	20q13.32	0,507576647	4,21E-28	8,24E-27
DMD	Xp21.2-p21.1	0,507482342	4,32E-28	8,45E-27
SPON2	4p16.3	0,507199268	4,68E-28	9,13E-27
ABCB4	7q21.12	0,50718637	4,70E-28	9,16E-27
LAMP5	20p12.2	0,50713463	4,76E-28	9,28E-27
SPTA1	1q23.1	0,507123476	4,78E-28	9,30E-27
COL18A1	21q22.3	0,507026115	4,91E-28	9,55E-27
CCL21	9p13.3	0,506590434	5,54E-28	1,08E-26
TLL1	4q32.3	0,506439968	5,78E-28	1,12E-26
PSTPIP1	15q24.3	0,506416017	5,82E-28	1,13E-26
NLRP12	19q13.42	0,505940334	6,64E-28	1,29E-26
PRND	20p13	0,50589671	6,73E-28	1,30E-26
RAB31	18p11.22	0,505755209	7,00E-28	1,35E-26
MGP	12p12.3	0,505609266	7,28E-28	1,41E-26
ITGA5	12q13.13	0,505347064	7,83E-28	1,51E-26
FAM30A	14q32.33	0,505293109	7,95E-28	1,53E-26

EGR3	8p21.3	0,505230274	8,09E-28	1,56E-26
CD302	2q24.2	0,505221616	8,11E-28	1,56E-26
PCDH12	5q31.3	0,505172851	8,22E-28	1,58E-26
ADAMTS9	3p14.1	0,505033026	8,55E-28	1,64E-26
TMEM100	17q22	0,504744114	9,26E-28	1,78E-26
ADAMTS16	5p15.32	0,504419188	1,01E-27	1,94E-26
KCNT2	1q31.3	0,504360957	1,03E-27	1,97E-26
TWIST2	2q37.3	0,504332884	1,04E-27	1,98E-26
DNAJB5	9p13.3	0,50412351	1,10E-27	2,10E-26
CSF3R	1p34.3	0,504049478	1,12E-27	2,14E-26
KCNJ5	11q24.3	0,503959083	1,15E-27	2,19E-26
DNM1P46	15q26.3	0,50373418	1,22E-27	2,33E-26
RAB42	1p35.3	0,503711653	1,23E-27	2,34E-26
TMEM130	7q22.1	0,503417186	1,34E-27	2,54E-26
LINC00092	9q22.32	0,503233889	1,41E-27	2,67E-26
MSN	Xq12	0,502786876	1,59E-27	3,01E-26
MEOX1	17q21.31	0,502778198	1,59E-27	3,02E-26
CACNA2D1	7q21.11	0,502730372	1,61E-27	3,06E-26
CLEC5A	7q34	0,502717107	1,62E-27	3,06E-26
COL4A1	13q34	0,502644467	1,65E-27	3,12E-26
TENM3	4q34.3-q35.1	0,502440467	1,75E-27	3,30E-26
DENND5B	12p11.21	0,502363712	1,79E-27	3,37E-26
RAB37	17q25.1	0,502232125	1,85E-27	3,49E-26
TSHZ3	19q12	0,501823581	2,07E-27	3,90E-26
SRPX	Xp11.4	0,50181139	2,08E-27	3,91E-26
BVES	6q21	0,501801849	2,08E-27	3,91E-26
FHL1	Xq26.3	0,501555195	2,23E-27	4,18E-26
CHST1	11p11.2	0,501548679	2,23E-27	4,19E-26
IL32	16p13.3	0,501431867	2,31E-27	4,32E-26
PGM2L1	11q13.4	0,501029552	2,57E-27	4,82E-26
MMP9	20q13.12	0,500983967	2,61E-27	4,87E-26
RCAN1	21q22.12	0,500638898	2,86E-27	5,35E-26
EMILIN2	18p11.32-p11.31	0,500204603	3,22E-27	6,02E-26
DPYSL3	5q32	0,500184461	3,24E-27	6,05E-26
NTM	11q25	0,50000053	3,41E-27	6,35E-26
SYTL1	1p36.11	-0,504433064	1,01E-27	1,93E-26

Figure S1

Immunohistochemical staining of IQGAP2 in NMIBC and metachronous MIBC
tabular form, continued on the next pages

	**NMIBC**	**MIBC**

patient 1

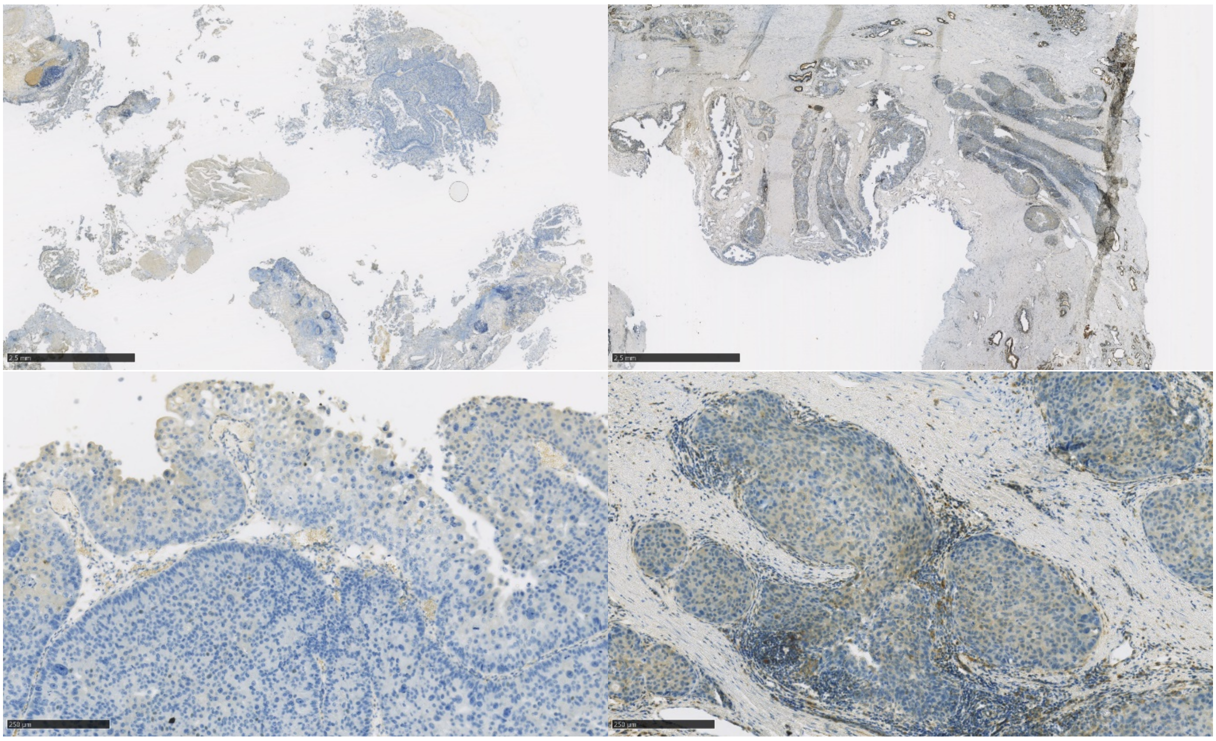

Only peripheral tumor cells are very weak stained.	Homogenously very weak stained tumor areas.

NMIBC **MIBC**

patient 2

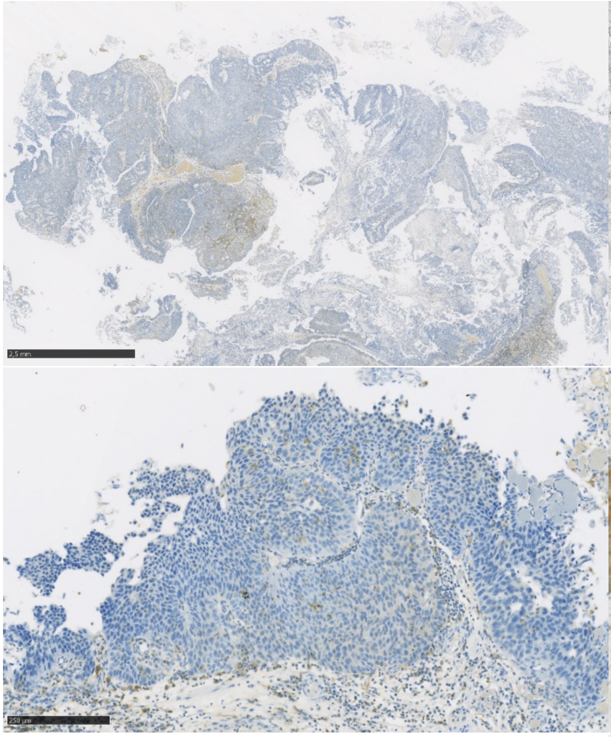

Negative, few single tumor cells are weak positive.

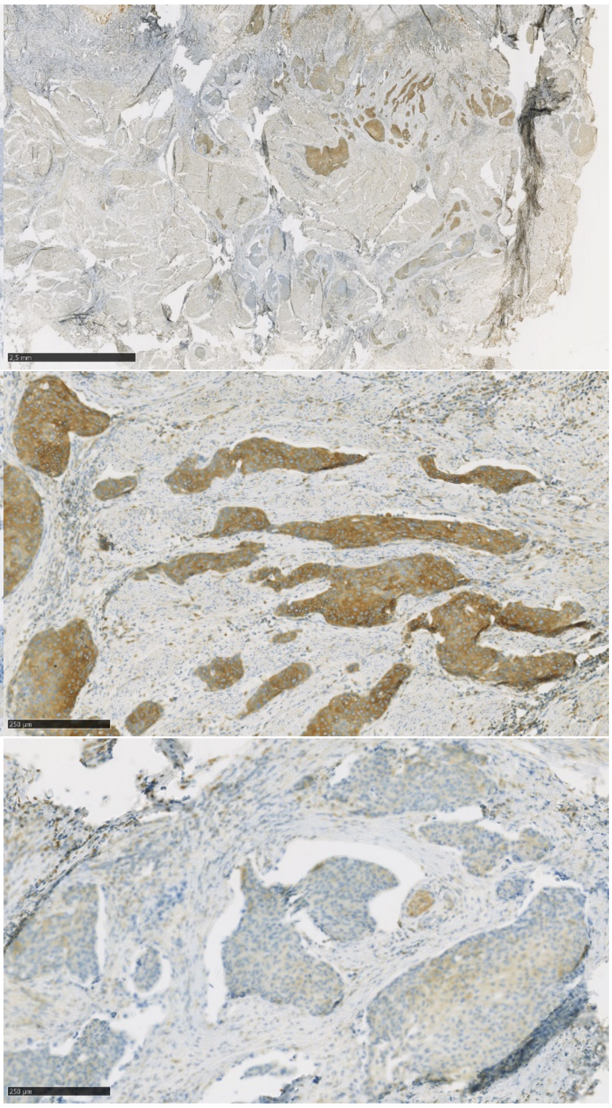

Negative, weak and strong stained tumor areas in equal ratios.

NMIBC **MIBC**

patient 4

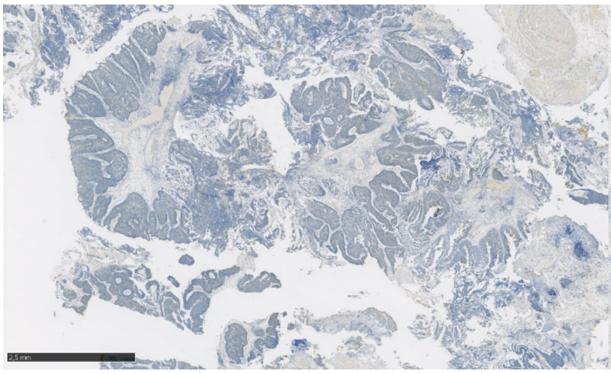

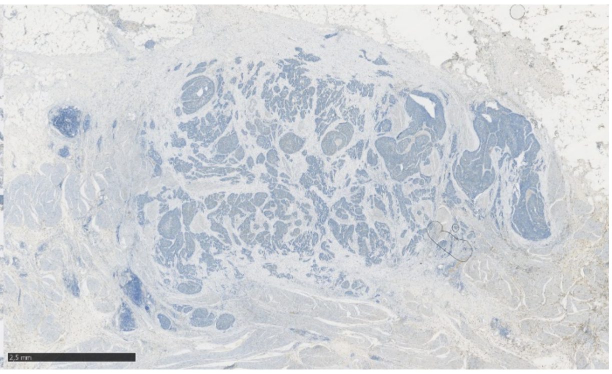

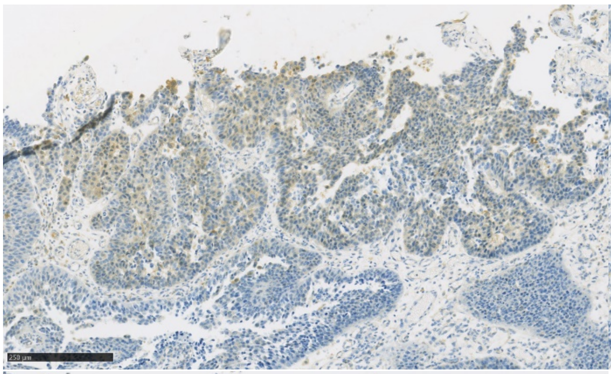

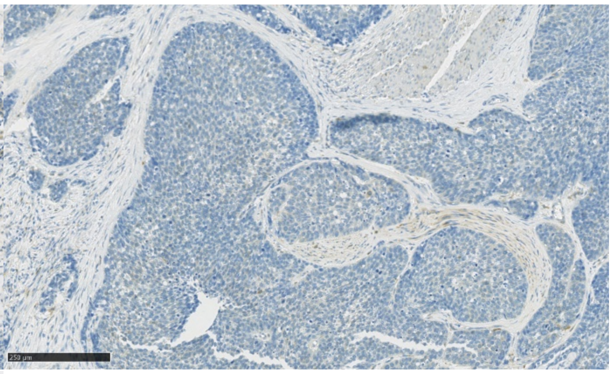

No staining or very weak staining of tumor cells.

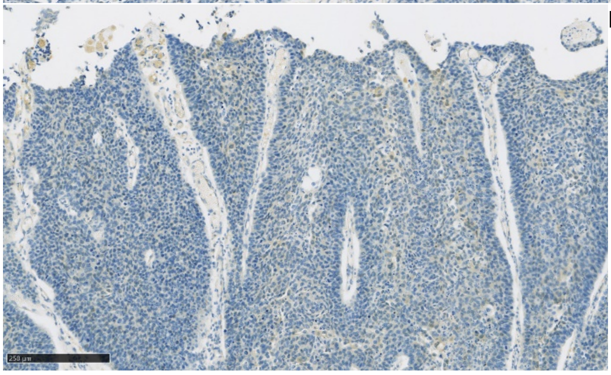

Some tumor areas are weak stained.

NMIBC **MIBC**

patient 5

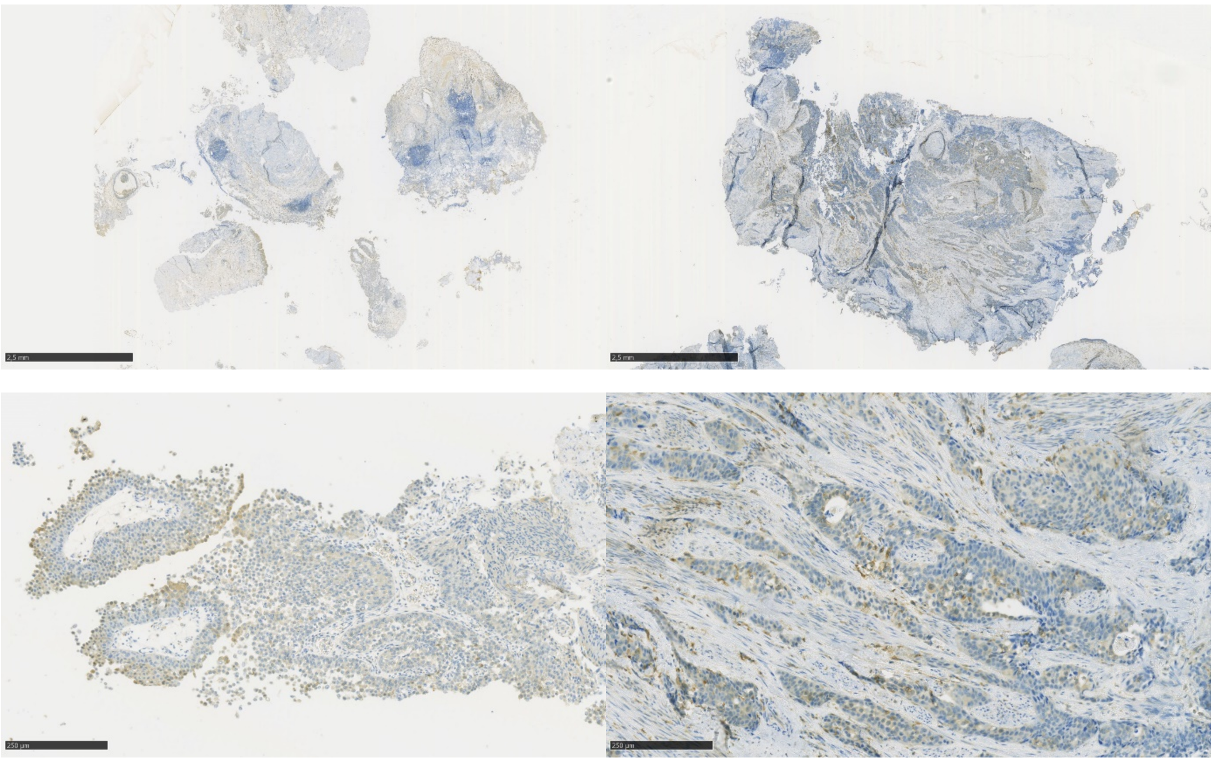

Weak to moderates staining.

Mainly negative to weak staining. Few strong stained single tumor cells.

NMIBC **MIBC**

patient 6

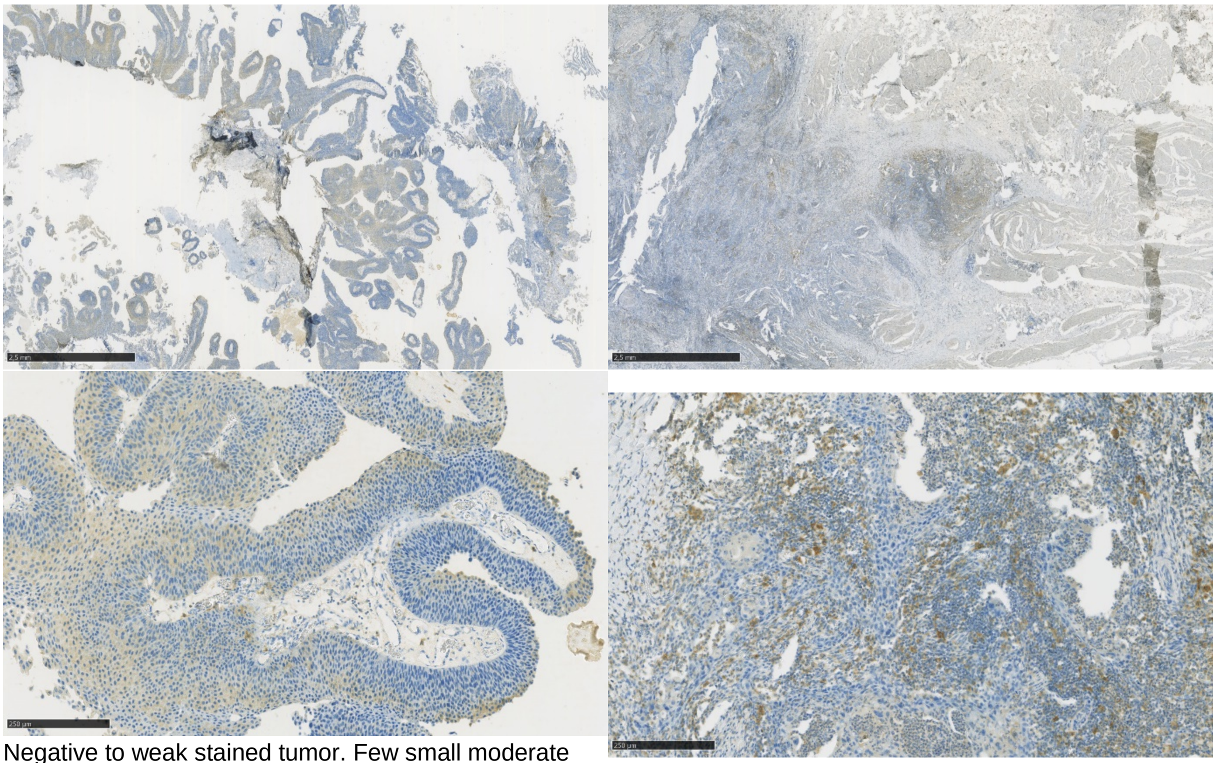

Negative to weak stained tumor. Few small moderate stained areas.

Negative as well as heterogeneous weak to moderate stained tumor cells. Few strong stained single tumor cells.

NMIBC | **MIBC**

patient 7

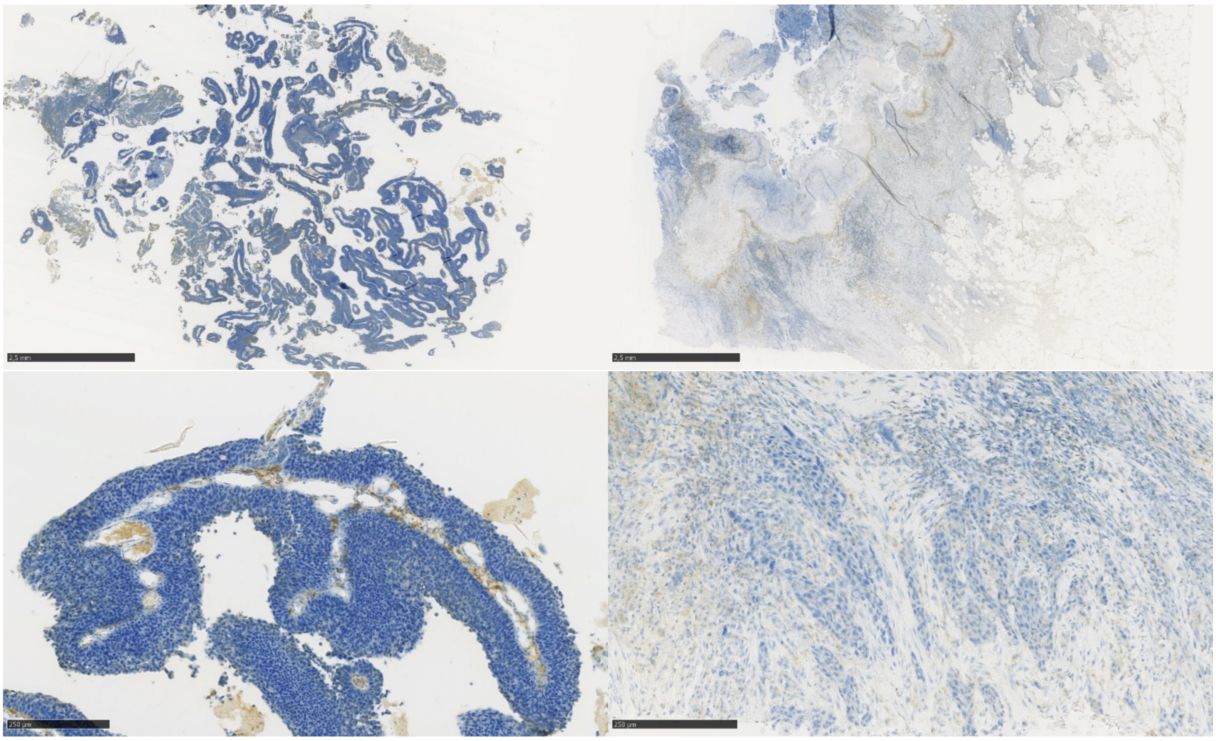

Mainly negative tumor areas. Focal weak stained peripheral tumor cells.

Mainly negative tumor areas. Few weak stained tumor cells.

NMIBC | **MIBC**

patient 8

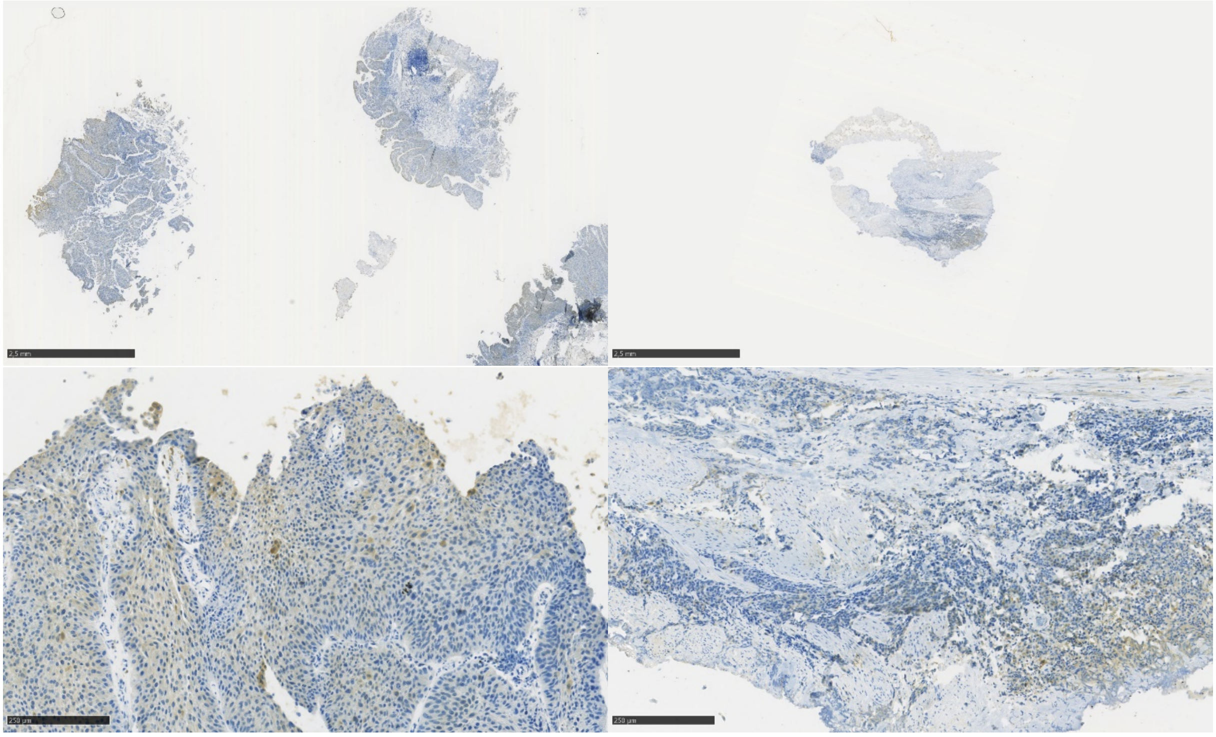

Negative to weak stained tumor cells. One small area with moderate stained peripheral cells.

Mainly negative tumor cells. Weak staining in area with artefacts (bottom right). May be not representative small sample.

Immunohistochemical staining of IQGAP2 in further MIBC (NMIBC not available)

MIBC

patient 3

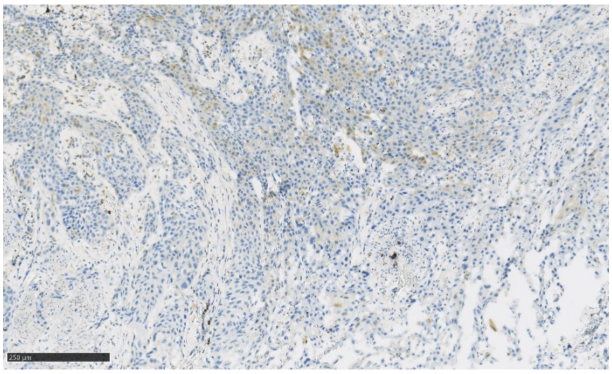

Negative tumor cells in muscle invasive layer. Increased weak staining of tumor cells towards non-muscle invasive areas and urothelium.

MIBC

patient 9

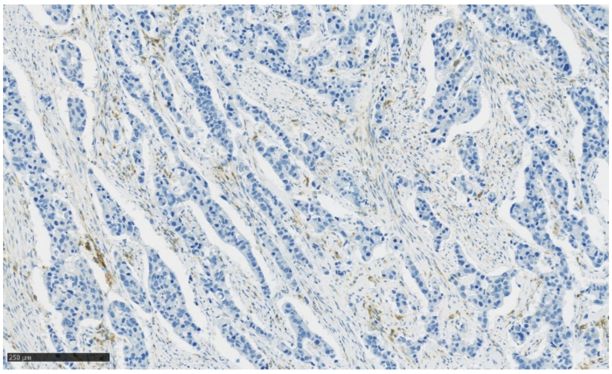

Completely negative tumor cells.

MIBC

patient 10

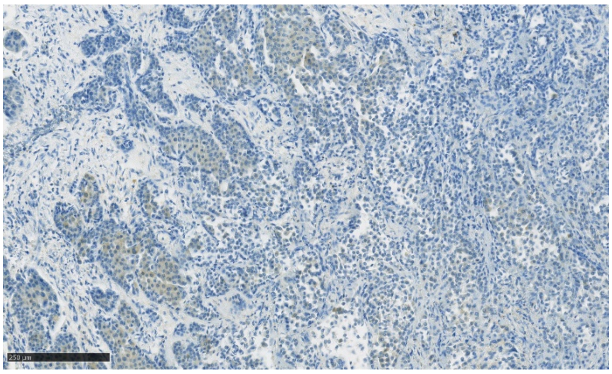

Mainly negative to weak and very small moderate stained tumor areas.

MIBC

patient 11

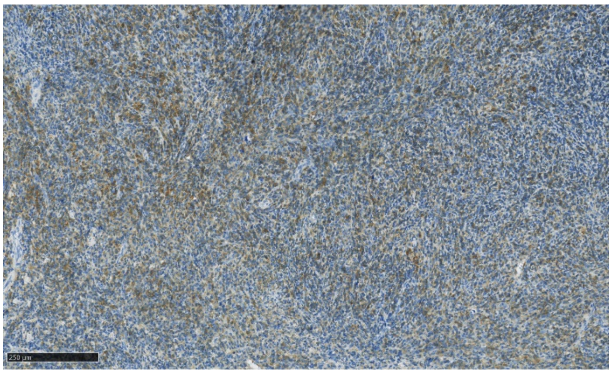

Tumor with strong infiltrated by immune cells. Partially weak to moderate stained tumor cells.

Immunohistochemical staining of IQGAP2 in dysplastic urothelium from tumor samples

dysplastic urothelium

patient 2

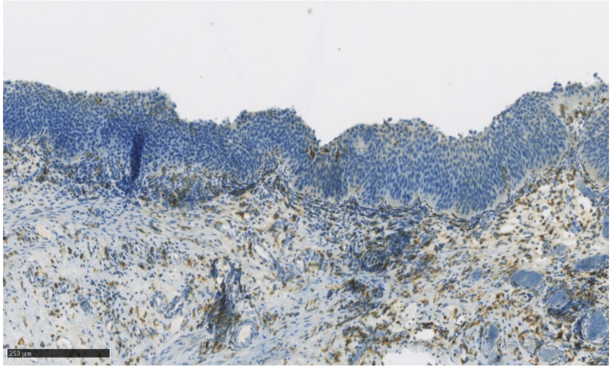

Mainly negative dysplastic urothelium with weak stained umbrella cells.

patient 3

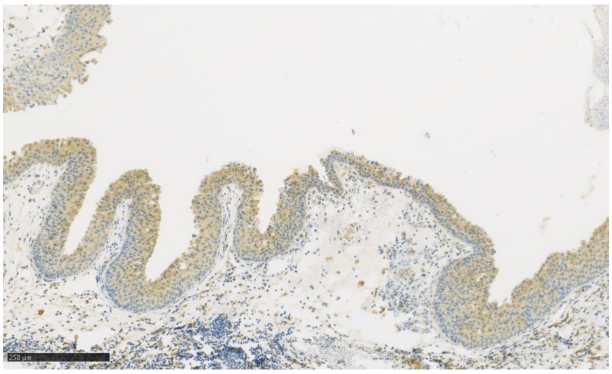

Weak to moderate stained dysplastic urothelium.

patient 6

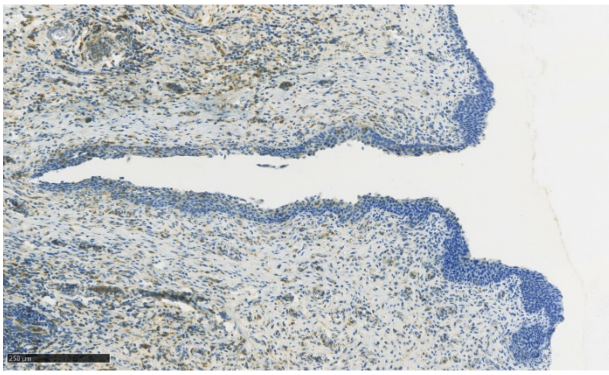

Negative dysplastic urothelium.

Immunohistochemical staining of IQGAP2 in normal-like non-dysplastic urothelium from tumor samples

normal-like (non-dysplastic) urothelium

patient 7

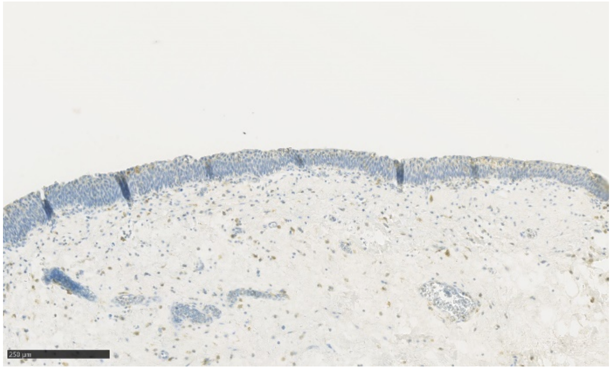

Mainly negative non-dysplastic urothelium with weak stained umbrella cells.

Patient 8

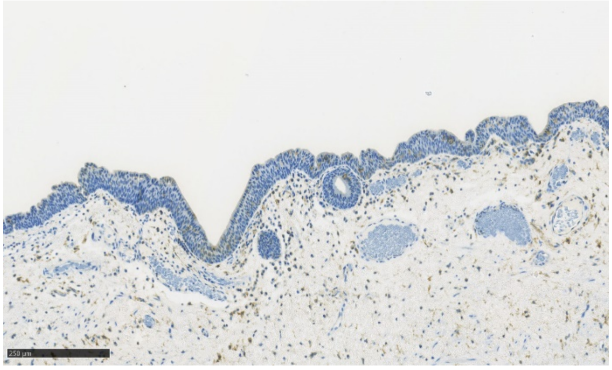

Mainly negative non-dysplastic urothelium with weak stained umbrella cells.

Supplementary figure 1. Immunohistochemical staining of BCL9L in NMIBC, MIBC, dysplastic urothelium and normal-like urothelium of patients with progressive Bladder Cancer. Overall average staining intensity of the tumor cells of the whole slide is specified as negative (-), weak (+), moderate (++) and strong (+++) referring to the proportion of tumor cells. Export from NDP.view as 1.25 or 10-fold magnification (2.5 mm/ 250 µm scale bar). Anti-IQGAP2 1:50, Santa-Cruz sc-17835

Figure S2

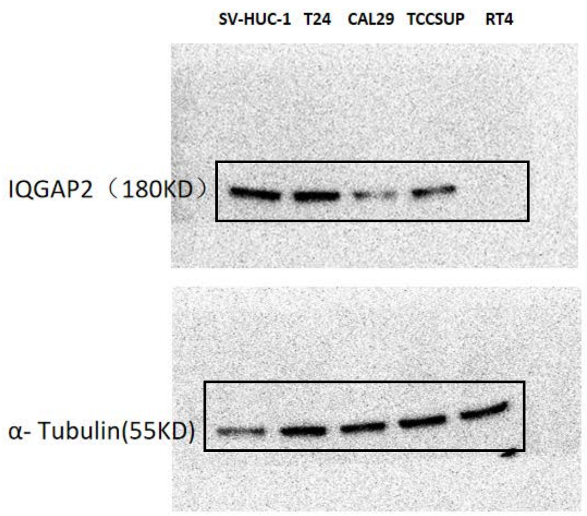

Supplementary figure 2.1. The IQGAP2 protein expression in 4 BC cell lines and 1 normal urothelial cell line

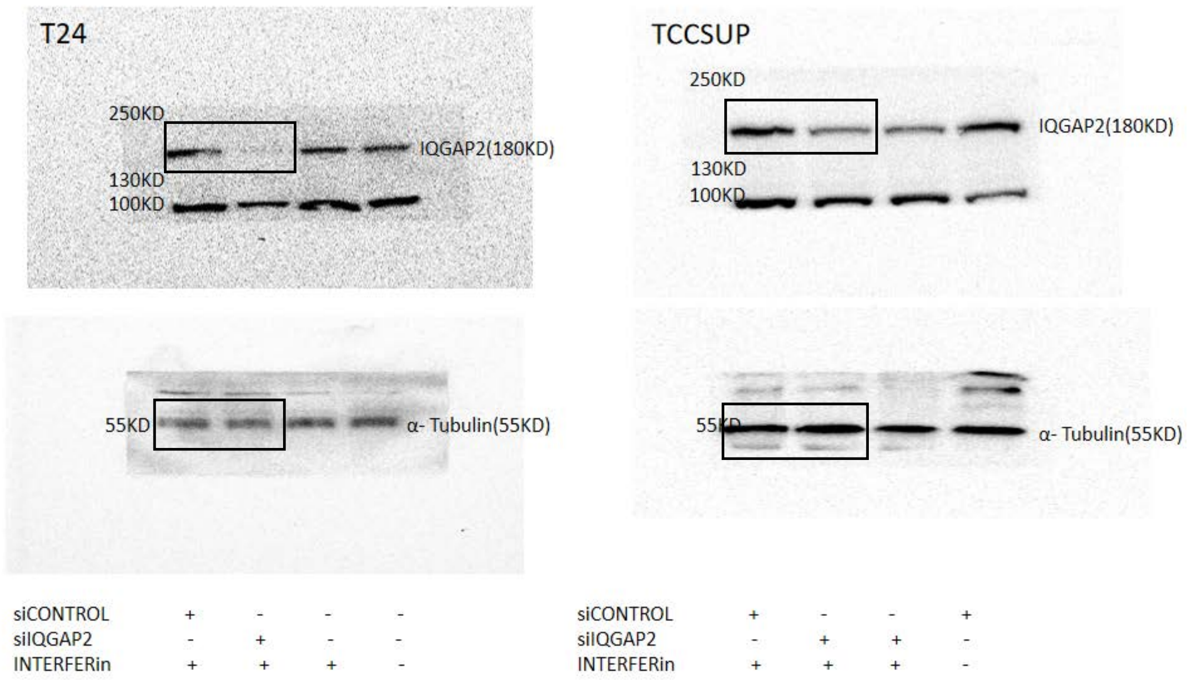

Supplementary figure 2.2. The knockdown confirmation of IQGAP2 in T24 and TCCsup cell line

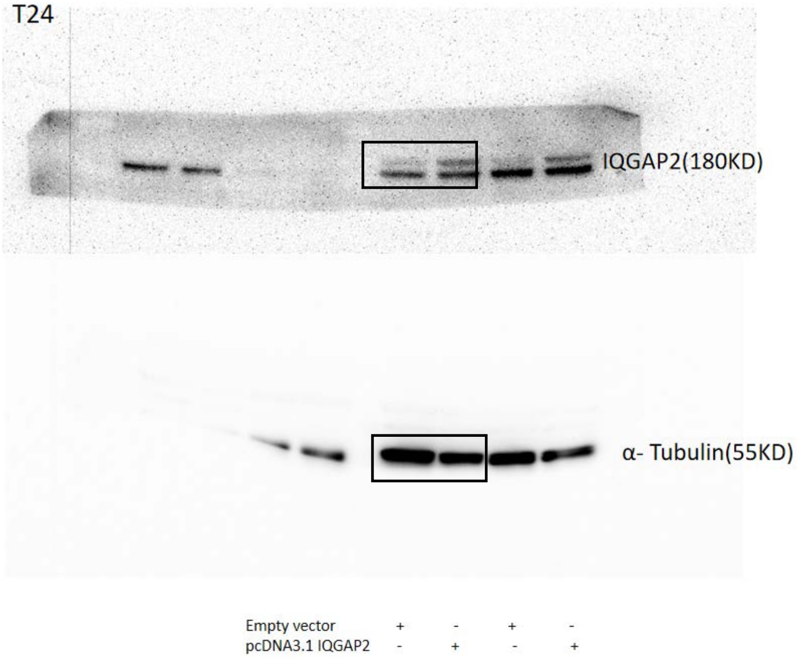

Supplementary figure 2.3. The ovexpression confirmation of IQGAP2 in T24 cell line

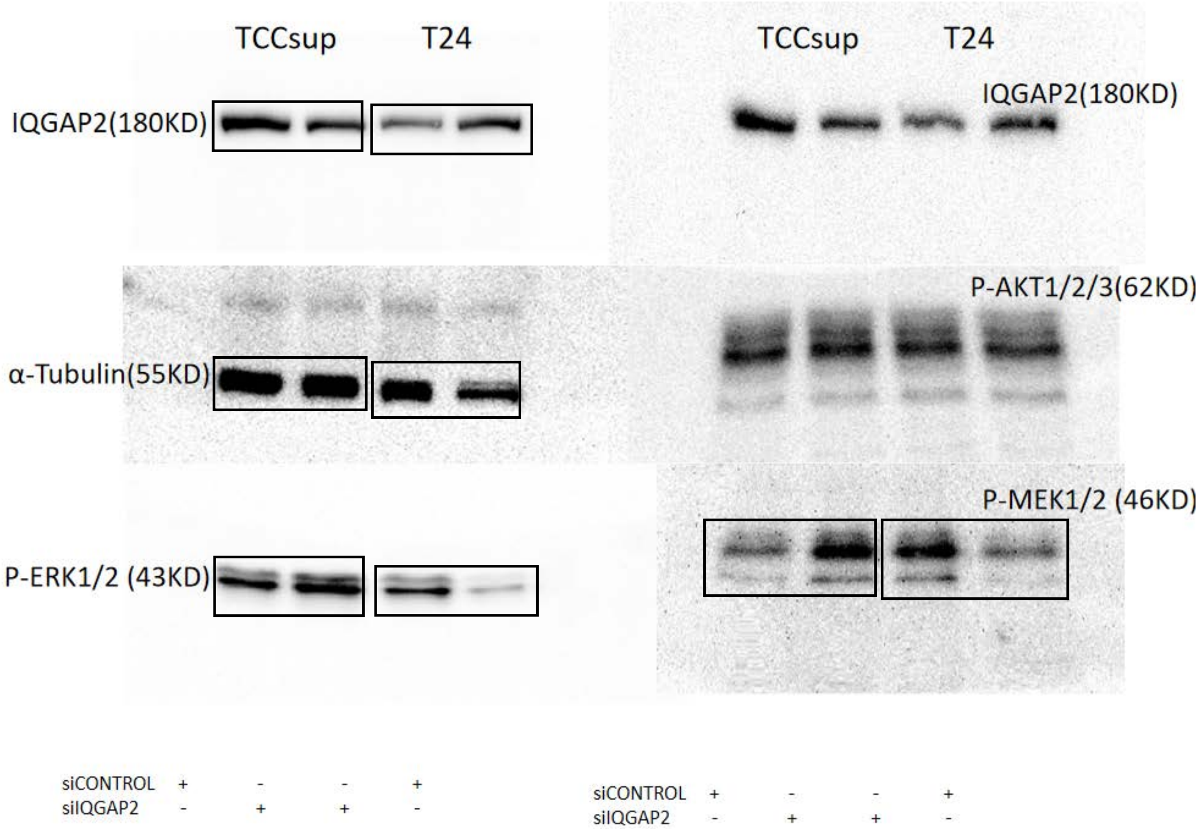

Supplementary figure 2.4. Depletion of IQGAP2 increased phospho-MEK and phospho-ERK1/2 in T24 and TCCSUP. Depletion of IQGAP2 didn't effect the expression of phospho-AKT1/2/3

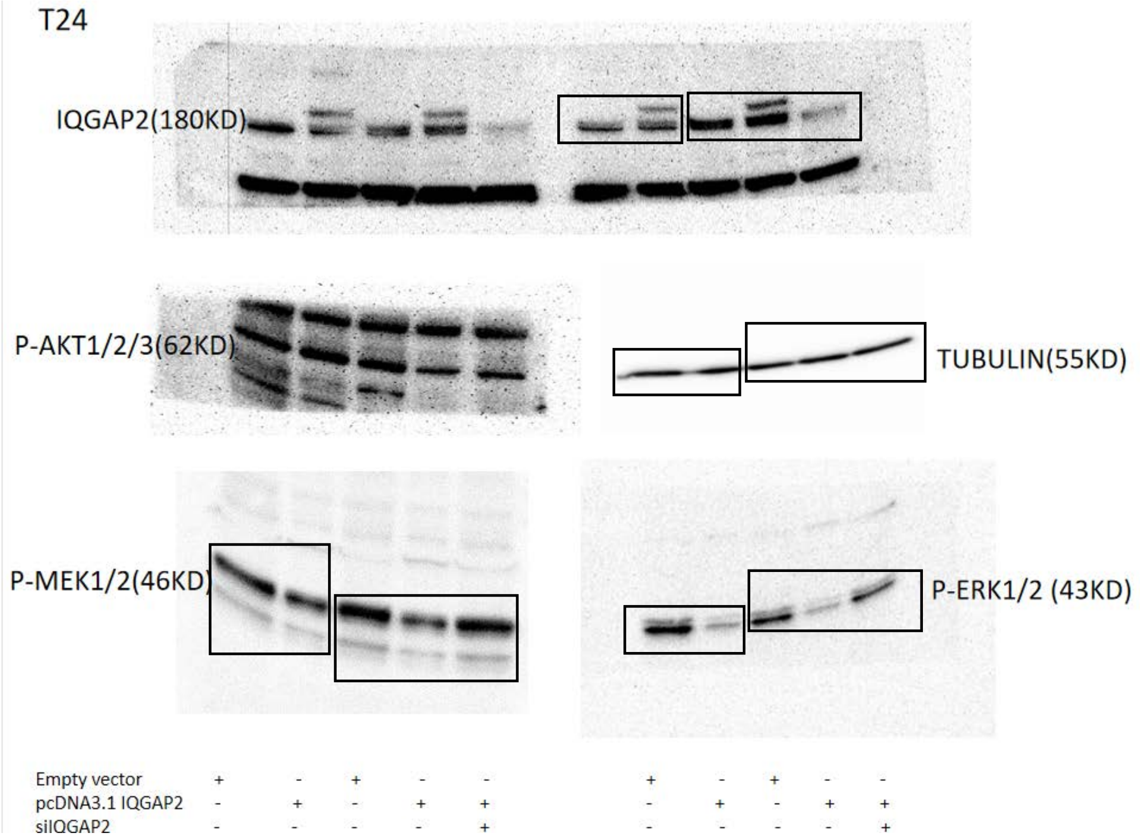

Supplementary figure 2.5. Overexpression of IQGAP2 decreased phospho-MEK and phospho-ERK1/2 in T24. Overexpression of IQGAP2 didn't effect the expression of phospho-AKT1/2/3

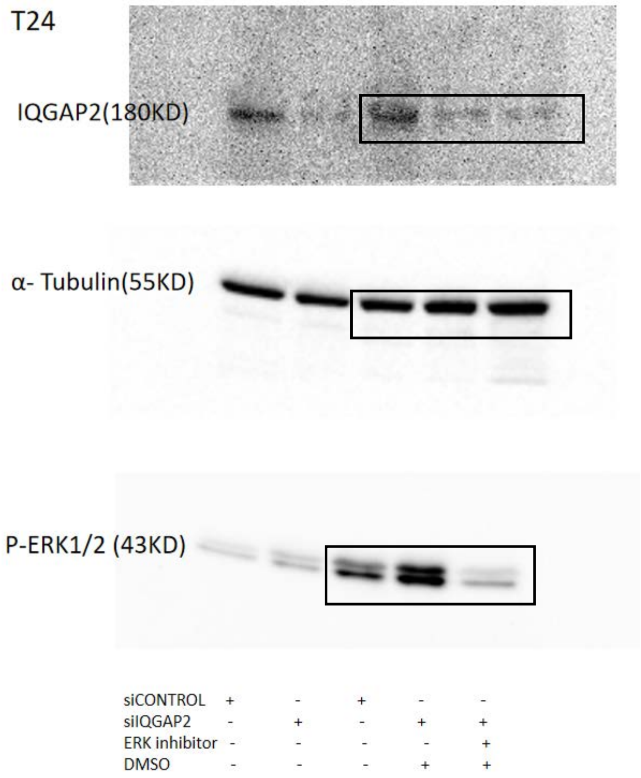

Supplementary figure 2.6. The MEK inhibitor U1026 rescued the activated p-ERK induced by IQGAP2 depletion in T24 cell line

Figure S3

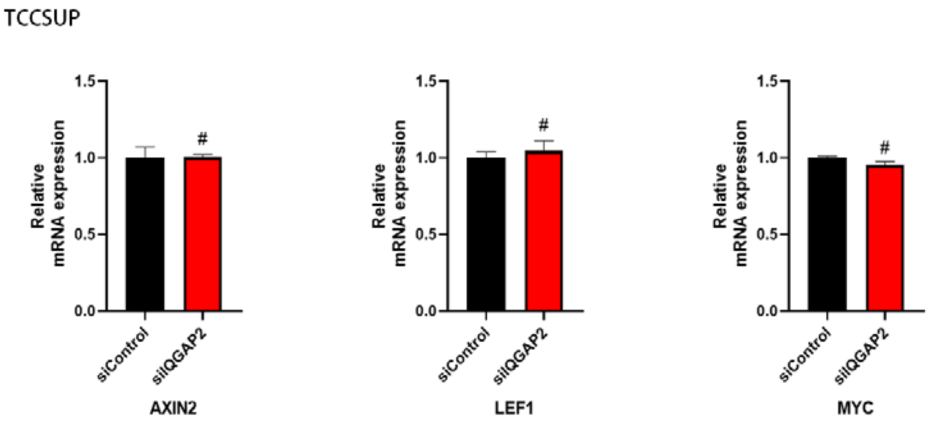

Supplementary figure 3. The depletion of IQGAP2 in TCCSUP didn’t effect the expression of wnt target genes
